# A theoretical analysis of mass scaling techniques

**DOI:** 10.1007/s00466-025-02611-7

**Published:** 2025-03-07

**Authors:** Yannis Voet, Espen Sande, Annalisa Buffa

**Affiliations:** https://ror.org/02s376052 grid.5333.6 0000 0001 2183 9049 MNS, Institute of Mathematics, École polytechnique fédérale de Lausanne, Station 8, CH-1015 Lausanne, Switzerland

**Keywords:** Mass scaling, Mass lumping, Explicit dynamics, Critical time step, Outlier removal

## Abstract

Mass scaling is widely used in finite element models of structural dynamics for increasing the critical time step of explicit time integration methods. While the field has been flourishing over the years, it still lacks a strong theoretical basis and mostly relies on numerical experiments as the only means of assessment. This contribution thoroughly reviews existing methods and connects them to established linear algebra results to derive rigorous eigenvalue bounds and condition number estimates. Our results cover some of the most successful mass scaling techniques, unraveling for the first time well-known numerical observations.

## Introduction and background

Finite element analysis (FEA) has long established itself as an indispensable tool in many engineering disciples. Particularly in structural dynamics, one of its founding applications, where the method is extensively used for simulating the deformation and vibration of various structures and solids, including plates, shells and beams. In this context, explicit time integration is favored in several circumstances. Indeed, the dynamics of the problem often impose a natural restriction on the step size [1], and enlarging it may lead to convergence issues within implicit unconditionally stable methods, specifically for nonlinear problems requiring the solution of a nonlinear system of equations at each time step. On the contrary, the smaller step size used within explicit methods ensures greater robustness. Moreover, explicit methods offer the possibility of “lumping” the mass matrix enabling memory-efficient matrix-free implementations. *Mass lumping* consists in directly substituting the mass matrix with an ad hoc diagonal approximation [2–5]. Thus, explicit methods may avoid solving costly linear or nonlinear systems of equations, let alone the burden of linearizing the stiffness.

Unfortunately though, explicit methods are only conditionality stable and the higher frequencies impose a strong restriction on the critical time step. For example, for undamped dynamical systems, the critical time step of the central difference method is

1.1 $$\begin{aligned} \Delta t_c= \frac{2}{\omega _n} \end{aligned}$$

where $\omega _n$ is the largest frequency of the discrete system [6, 7]. The largest discrete frequencies of classical $C^0$ finite element methods are wholly inaccurate and fortunately rarely contribute significantly to the solution. They form the so-called “optical branches” of the spectrum and diverge with the mesh size and polynomial degree [8, 9]. Smooth isogeometric analysis (IGA) [10] features far fewer inaccurate frequencies [11–14]. They usually form noticeable spikes in the upper part of the spectrum and were coined *outliers* for this very reason [11]. Regardless of the discretization technique and smoothness, large inaccurate eigenvalues severely restrict the critical time step and removing or dampening them is paramount in explicit dynamics. The method for removing them, however, usually depends on the discretization technique. In isogeometric analysis, so-called *outlier removal techniques* commonly exploit the smoothness and tensor product nature of spline spaces (see e.g. [11, 14–19]). These methods may also preserve the consistency of the discrete formulation and therefore higher rates of convergence. In more general cases with significantly less structure, one may resort to *mass scaling*. Broadly speaking, mass scaling consists in dampening higher frequencies by altering the discrete formulation and, more specifically, the mass matrix. *Conventional mass scaling* (CMS) [20, 21], which only scales the diagonal entries of the mass matrix, is the most straightforward way of achieving this goal. Unfortunately, it also heavily deteriorates the accuracy of the lower frequencies and mode shapes, and, subsequently, the numerical solution. *Selective mass scaling* (SMS) is specifically designed for scaling down the highest frequencies while somewhat preserving the lowest ones. Thus, it still affects *all* eigenvalues, but not uniformly. Mathematically speaking, the idea is reminiscent of inexact deflation techniques, which consist in removing unwanted eigenvalues from the spectrum of a matrix. Exact deflation techniques have been known for decades and originated from the early work of Hotelling [22] for standard eigenproblems but are mostly impractical. Practical strategies, either global or local, are instead essentially heuristic and oftentimes little is known of their theoretical properties. Global strategies directly modify the assembled system matrices while local ones operate on the element matrices prior to assembly. The former are generally easier to analyze than the latter and are among the oldest methods. In the late 1990s, Macek and Aubert [23] and later Olovsson et al. [1] independently proposed the same global mass scaling procedure, based on linear fractional transformations of matrix pairs. The method was later generalized in [24] to rational polynomials, where the authors also stated optimality requirements for SMS. These methods are fully understood and transform (nonlinearly) the eigenvalues of a matrix pair while preserving its eigenvectors. Although the methods affect all eigenvalues, they may heavily damp the largest ones, while nearly preserving the smallest ones. In the same article, the authors also proposed an exact deflation method only scaling down the largest eigenvalues of the system. The idea was later revived and thoroughly improved in [25] but remains impractical, unless only a few well-separated eigenvalues must be deflated [19]. As we will see, all of these methods are merely adaptions or reformulations of well-known linear algebra facts, mostly known for decades.

Since the early 2000s, focus has slowly shifted towards inexact global or local strategies. Global strategies often have a local counterpart, which simply consists in performing the same operations locally prior to assembly. Local strategies naturally suggest themselves when local properties dictate global ones. For instance, for hexahedral elements, the largest eigenvalues are tied to large aspect ratios [26–28] while for beam and plate models, they are associated to transverse shear modes [29–31]. Already in the 1970s, several authors suggested locally scaling rotational components of lumped mass matrices within beam models [20, 21]. This technique, nowadays known as *rotational mass scaling* (RMS), merely applies conventional mass scaling to selected degrees of freedom and is rather inaccurate [30, 31]. As shown by Oesterle et al. [29], its accuracy is sometimes significantly improved by simply reparameterizing the model and choosing different primary unknowns. This concept of *intrinsically selective mass scaling* (ISMS) was recently extended to plate formulations [31].

For hexahedral elements, Olovsson et al. [26] first suggested grouping nodes aligned in the thickness direction of thin-walled structures and locally scaling the inverse mass matrices of each individual group. A rather similar strategy was followed by Cocchetti et al. for non-distorted [27] and distorted [28] hexahedral elements. Also in the early 2000s, Olovsson et al. [1] suggested instead locally scaling the element mass matrices of hexahedral finite elements and highlighted some appealing numerical properties. Nowadays, the method, together with a miscellaneous collection of variants and generalizations [32, 33], is strongly rooted in the engineering community and has been incorporated in commercial finite element software for car-crash simulations, such as LS-DYNA [33] and RADIOSS [34]. Several improvements have also lately been suggested in [35]. As we will see, some of these methods are (inexact) local deflation techniques. Exact local versions were investigated in [19, 24, 25, 36, 37], sometimes by simply adapting their global counterpart. These methods are similar to eigenvalues stabilization techniques [38], which are one of numerous ad hoc stabilization schemes for immersed methods [39, 40].

A completely different approach was followed in [41] where the authors derived a parametrized family of mass matrices from a penalized Hamilton’s principle. Interestingly, the method of Olovsson et al. may be recovered (up to a multiplicative factor) for specific parameter values and Ansatz spaces. However, the authors propose scaling the consistent mass matrix and the subsequent improvement of the critical time step may not even reach beyond the one for a lumped mass matrix. Additionally, the authors report severe conditioning issues, sometimes even preventing the convergence of iterative solvers. To our knowledge, this method has not been widely adopted in practice. In [42, 43], the authors used a similar strategy for building directly a sparse approximate inverse of the consistent mass (therein referred to as *reciprocal mass matrix* (RMM)) and a variationally scaled version thereof. While this strategy alleviates the burden of solving linear systems with the scaled mass matrix, it also does not always increase the critical time step with respect to the lumped mass matrix. Moreover, the definiteness of the scaled approximate inverse practically limits the range of parameter values and prevents increasing the step size beyond 50%. Finally, technical issues tied to dual polynomials further impede the method, such as the imposition of Dirichlet boundary conditions. A simpler alternative based on Lagrange multipliers was proposed for FEA [37] and later extended to IGA [44].

Performing operations locally is often significantly faster than globally and offers potential for parallelization. However, while some global methods may be classified as “exact”, most local methods rarely are due to the assembly process. Quantifying and analyzing their “inexactness” is one of the objectives of this article. Before that, the heterogeneous collection of methods listed above asks for a thorough review. Among the aforementioned mass scaling techniques, we have identified three common shortcomings:

1. Most practically relevant methods are inexact and will surely affect the smallest eigenfrequencies. The accuracy of the methods is often only verified by comparing them to the solution with a lumped mass matrix, which might not always yield an accurate approximation. Additionally, mass scaling may reduce the convergence rate of the smallest eigenvalues, if not already ruined by mass lumping. To our knowledge, few authors have undertaken a convergence study for their method.
2. In addition to sometimes (critically) affecting the accuracy, mass scaling often leads to a non-diagonal scaled mass matrix, thereby partly undermining the computational savings from the reduction in the number of time steps. Surprisingly few authors have investigated this tradeoff and the time savings (if any) might come at the price of increased storage requirements. Some of the methods proposed are impractical and may not compete with a lumped mass, leaving alone accuracy concerns. Due to the non-diagonal scaled mass, researchers have resorted to preconditioned iterative methods [24, 45] or Cholesky factorizations [40, 46] for solving linear systems. The former has also driven condition number analyses for the scaled mass.
3. Mass scaling methods are first and foremost designed to increase the critical time step but theoretical bounds on the step size are rarely provided. Some heuristic and “analytical” step size estimates were derived for FEA [27, 28] and IGA [47, 48], often by means of characteristic lengths. However, they generally do not guarantee a stable step size [49], unless derived from rigorous bounds based e.g. on the Gershgorin circles [49–51] or on Ostrowski’s bound [52].

In view of the points raised above, our article complements previous numerical studies with a theoretical analysis, which we feel is needed. Our analysis offers a unifying picture and a clearer positioning of the main methods proposed in the literature and is substantiated with some eigenvalue, step size and condition number estimates. Our study focuses exclusively on theoretical aspects and sheds light on the numerical observations reported in the literature. The few numerical experiments of this article are only meant to validate the theoretical predictions. Although mostly developed and applied for $C^0$ discretizations, some of the methods discussed herein are also valuable for smooth spline discretizations (i.e. IGA) and fit in the framework of outlier removal techniques provided one substitutes elements for patches.

The outline of the article is as follows: in Section  2, we first formally introduce mass scaling as an eigenvalue perturbation problem and also recall the terminology adopted within the community. Our analysis then focuses on global (Section  2.1) and local (Section  2.2) techniques. In both sections, we first recall some general results in eigenvalue perturbation theory before establishing rigorous bounds on the eigenvalues, step size, and condition number. Our bounds prove some of the observations stated by the original authors and support their numerical findings. Finally, Section  3 presents a few numerical experiments highlighting the sharpness of our bounds and provides further insights on the behavior of the methods. Lastly, Section  4 summarizes our findings and suggests improvements for future work.

## Mass scaling

Mass scaling consists in perturbing the mass matrix in order to drive down the largest generalized eigenvalues constraining the critical time step of explicit time integration schemes, while ideally leaving the smallest ones unaffected. In general, the scaled mass matrix $\overline{M}$ is obtained by adding to the (lumped) mass matrix *M* a well chosen symmetric positive semidefinite perturbation *E*:

$$\begin{aligned} \overline{M}=M+E. \end{aligned}$$

Scaled quantities are often denoted with an overline and we will stick to this convention. Since we are exclusively dealing with real quantities, it will not be misidentified with complex conjugacy. Oftentimes, the mass matrix *M* is lumped [1, 27, 29, 41] with any suitable strategy such as the row-sum technique [6] or the HRZ (Hinton-Rock-Zienkiewicz or diagonal scaling) method [53]. The scaling matrix *E* is sometimes only defined locally, for each finite element, and the global matrix is then obtained from the usual assembly of element contributions. The success of mass scaling lies in the definition of *E*, which often depends on the problem and its discretization. The strategies proposed commonly fall in either one of the following categories:

- *Conventional mass scaling* (CMS), also sometimes called *regular mass scaling*, only scales the diagonal entries of the mass matrix. It is called *uniform* when all diagonal entries are scaled uniformly. Although it preserves the diagonal structure of the lumped mass matrix, it also scales down *all* eigenvalues and adversely affects the dynamics, unless performed locally on critical elements within non-uniform meshes that severely constrain the step size [26–30, 35]. CMS is also typically used for scaling rotational degrees of freedom of beam or shell finite element formulations [20, 21]. Despite being oftentimes inaccurate, this strategy is still widely used in commercial software packages, such as LS-DYNA [54]. Nevertheless, as shown in [29, 31], simply reformulating the original model may sometimes improve the accuracy.
- *Selective mass scaling* (SMS), instead, selectively scales down the highest eigenfrequencies whose mode shapes are oftentimes described as structurally “irrelevant” [29, 30]. Unfortunately, SMS usually leads to non-diagonal scaled mass matrices and requires solving a linear system at each time step. Not only does it ruin the fundamental concept of explicit time integration but it also requires a careful assessment of the tradeoff between the reduction of the number of time steps and the increased cost per time step. Yet, the most successful mass scaling strategies fall in this second category and will be at the center of our attention in the forthcoming discussion.

As mentioned earlier, mass scaling strategies may be defined globally or locally. Global strategies often have appealing spectral properties but are rather impractical. Local strategies, instead, are more convenient and are usually favored for $C^0$ finite element discretizations, especially for beam, plate and shell formulations featuring both translational and rotational degrees of freedom (see e.g. [20, 21, 55]). However, their analysis is more tedious. In the sequel, we will distinguish these two cases as their treatment is fundamentally different.

### Global mass scaling

#### Preliminaries

In this section, we assume that the perturbation $E \in \mathbb {R}^{n \times n}$ is defined globally; i.e. its construction is oblivious to the underlying finite element method. Mass scaling, in the most general and abstract terms, may be seen as an eigenvalue perturbation problem. That is, given a symmetric matrix pair (*A*, *B*) with *B* positive definite, we must analyze how far off the eigenvalues of $(A,B+E)$ are from those of (*A*, *B*). The perturbation theory for generalized eigenproblems is the object of a rich literature, which has flourished ever since the groundbreaking work of Stewart in the late 1970s [56]. However, most of the perturbation bounds are ill-suited for describing the properties of SMS since they bound some error measure of the eigenvalues *uniformly* (i.e. independently of the eigenvalue number) whereas SMS predominantly perturbs selected eigenvalues. Qualitatively speaking though, the bounds indicate that small eigenvalues are less sensitive to perturbations than larger ones and might provide some early insight.

Instead of treating *E* as some random perturbation, we will review some of the actual strategies proposed in the literature and position them in a more general context. It turns out none of the methods reviewed in this section are fundamentally new. As a matter of fact, their theoretical foundations were already laid out decades ago [56–58]. We first provide some definitions and recall a few fundamental properties of generalized eigenproblems. Throughout this article, we consider the set of pairs of matrices $V:= \mathbb {R}^{n \times n} \times \mathbb {R}^{n \times n}$, endowed with an obvious vector space structure:

- $$\forall (A,B),(C,D) \in V$$,
  $$\begin{aligned} (A,B)+(C,D)=(A+C,B+D). \end{aligned}$$
- $$\forall (A,B) \in V$$, $$\forall \mu \in \mathbb {R}$$,
  $$\begin{aligned} \mu (A,B) = (\mu A, \mu B). \end{aligned}$$

In other words, $V:= \mathbb {R}^{n \times n} \times \mathbb {R}^{n \times n}$ may be identified with $\mathbb {R}^{n \times 2n}$, to which it is isomorphic. This identification allows performing operations on matrix pairs as if they were block matrices. Moreover, we will exclusively focus on symmetric pairs (i.e. pairs formed by symmetric matrices) and in this context there exists a natural (partial) ordering.

##### Definition 2.1

(Loewner partial order) For two symmetric matrices $A,B \in \mathbb {R}^{n \times n}$, we write $A \succeq B$ (respectively $A \succ B$) if $A-B$ is positive semidefinite (respectively positive definite).

For convenience, we also define the sets of positive (semi-) definite matrices.

##### Definition 2.2

The sets of symmetric positive semidefinite (SPSD) and symmetric positive definite (SPD) matrices of size *n* are defined, respectively, as

$$\begin{aligned} \mathcal {S}_n= &   \{B \in \mathbb {R}^{n \times n} :B=B^T, B \succeq 0\} \quad \text {and} \quad \\ \mathcal {S}_n^+= &   \{B \in \mathbb {R}^{n \times n} :B=B^T, B \succ 0\}. \end{aligned}$$

The analysis conducted in this article pertains to generalized eigenvalues and eigenvectors of matrix pairs. A pair $(\lambda , \textbf{u}) \in \mathbb {C} \times \mathbb {C}^n$ with $\textbf{u} \ne \textbf{0}$ is called a generalized eigenpair of (*A*, *B*) if

$$\begin{aligned} A \textbf{u} = \lambda B \textbf{u}. \end{aligned}$$

This definition is called the *standard form* of the eigenvalue problem. In contract, the mathematical literature commonly adopts the *cross-product form*, whereby a pair $(\alpha , \beta ) \in \mathbb {C}^2$ is called an eigenvalue if there exists $\textbf{u} \ne \textbf{0}$ such that

$$\begin{aligned} \beta A \textbf{u}=\alpha B \textbf{u}. \end{aligned}$$

This representation treats *A* and *B* symmetrically. The corresponding generalized eigenvalue in standard form is recovered as $\lambda = \frac{\alpha }{\beta }$ (if $\beta \ne 0$ and is infinite otherwise). Obviously, this representation is non-unique since

2.1 $$\begin{aligned} (\alpha ,\beta ) \quad \text {and} \quad (t \alpha , t \beta ), \quad t \ne 0 \end{aligned}$$

refer to the same eigenvalue. Thus, rigorously speaking, one must identify an eigenvalue with an equivalence class (see e.g. [57]). Generalized eigenproblems exhibit some unusual behaviors, which we ought to prevent.

##### Definition 2.3

A matrix pair $(A,B) \in V$ is called *singular* if

$$\begin{aligned} \det (\beta A - \alpha B)=0 \end{aligned}$$

for all $(\alpha ,\beta ) \in \mathbb {C}^2$ and is called *regular* otherwise.

For a singular pair (*A*, *B*), a vector $\textbf{u} \in \ker (\beta A - \alpha B)$ is strictly speaking an eigenvector associated to an *arbitrary* eigenvalue $(\alpha ,\beta )$. Singular matrix pairs are pathological to say the least. Symmetric pairs are not necessarily regular and even if they are, contrary to standard eigenproblems, their generalized eigenvalues are in general complex (see e.g. [59, 60]). However, if there exists $(\alpha ,\beta ) \ne (0,0)$ such that $\det (\beta A - \alpha B) \ne 0$, then the pair (*A*, *B*) is regular. This is in particular the case if one of the matrices is invertible (take $(\alpha ,\beta )=(1,0)$ or $(\alpha ,\beta )=(0,1)$). Thus, a symmetric pair consisting of at least one positive definite matrix is regular. This condition is also sufficient for guaranteeing real eigenvalues [57, Theorem VI.1.15]. The next lemma provides a slightly stronger condition, which is always satisfied in our applications. The proof is an immediate extension of the aforementioned theorem.

##### Lemma 2.4

([57, Theorem VI.1.15]) Let $(A,B) \in \mathcal {S}_n \times \mathcal {S}_n^+$. Then, all generalized eigenvalues of (*A*, *B*) are real nonnegative and there exists an invertible matrix $U \in \mathbb {R}^{n \times n}$ such that

$$\begin{aligned} U^TAU=D, \qquad U^TBU=I, \end{aligned}$$

where $D={{\,\mathrm{\operatorname {diag}}\,}}(\lambda _1, \dots , \lambda _n)$ is a real nonnegative diagonal matrix containing the eigenvalues.

It can easily be shown that all eigenvalues are additionally positive if $A \in \mathcal {S}_n^+$. This condition will often be fulfilled in the forthcoming analysis. To avoid potential confusion, we will often specify the matrix pair when referring to eigenvalues, which are commonly numbered in ascending algebraic order; i.e.

$$\begin{aligned} \lambda _1(A,B) \le \lambda _2(A,B) \le \dots \le \lambda _n(A,B). \end{aligned}$$

The set of all eigenvalues (i.e. the spectrum) will be denoted $\Lambda (A,B)$. Furthermore, in our context, we define an *eigenfrequency* as $\omega = \sqrt{\lambda }$. The next lemma is the main building block for mass scaling techniques.

##### Lemma 2.5

([4, Corollary 3.6]) Let $A \in \mathcal {S}_n$, $B,\overline{B} \in \mathcal {S}_n^+$ and denote $E=\overline{B}-B$. Then the statements

1. $$E \succeq 0$$,
2. $$\overline{B} \succeq B$$,
3. $$\Lambda (B,\overline{B}) \subset (0,1]$$,

are all equivalent and imply that $\lambda _k(A,\overline{B}) \le \lambda _k(A,B)$ for all $k=1,\dots ,n$.

Thus, in particular, positive semi-definiteness of the scaling matrix *E* guarantees a decrease of the generalized eigenvalues. This is usually the first prerequisite in designing mass scaling techniques.

#### Linear fractional transformations

Modifying a matrix pair generally modifies its eigenvalues nontrivially. However, there exist special transformations for which the transformed eigenvalues are known explicitly. Linear Fractional Transformations are one of them.

##### Definition 2.6

(Linear factional transformation) A linear application $L :V \rightarrow V$ defined as

$$\begin{aligned} L(A,B):= (w_{11}A+w_{21}B, w_{12}A+w_{22}B) \end{aligned}$$

where $w_{ij} \in \mathbb {R}$ for $i,j=1,2$ is called a *linear fractional transformation* (LFT).

LFTs are analogous to shift-and-invert strategies [59, 60] and are widely used within eigensolvers for accelerating the convergence to desired eigenvalues. In this context, they are better known as *spectral transformations* [61]. The upcoming lemma and subsequent remark will better explain the name given to this type of transformation.

##### Remark 2.7

As noted in [57], by gathering the coefficients $w_{ij}$ in a matrix $W \in \mathbb {R}^{2 \times 2}$, the application may be compactly expressed as

$$\begin{aligned} L(A,B)=(A,B)(W \otimes I_n) \end{aligned}$$

and is entirely determined by the matrix *W*.

##### Definition 2.8

(Non-degenerate LFT) An LFT is called *degenerate* if $\det (W)=0$ and is called *non-degenerate* otherwise.

As shown in the following technical lemma ( [57, Theorem VI.1.6] and [60, Theorem 9.1]), LFTs preserve the eigenvectors and transform the eigenvalues in a simple manner. We include its proof, which is an exercise in [57].

##### Lemma 2.9

Let $(A,B) \in V$ be a regular matrix pair and *L*(*A*, *B*) be a non-degenerate LFT. Then,

- The eigenvectors of (*A*, *B*) and *L*(*A*, *B*) are the same.
- The eigenvalues $(\alpha ,\beta )$ of (*A*, *B*) are related to the eigenvalues $(\overline{\alpha },\overline{\beta })$ of *L*(*A*, *B*) through the relation
  $$\begin{aligned} (\overline{\alpha },\overline{\beta }) = (\alpha ,\beta )W. \end{aligned}$$

##### Proof

Let $(\alpha ,\beta )$ be an eigenvalue of (*A*, *B*) associated to an eigenvector $\textbf{u}$. The eigenvalue equation $\beta A \textbf{u}=\alpha B \textbf{u}$ can be expressed as $(A,B)(\textbf{v} \otimes \textbf{u})=0$ where $\textbf{v}^T=(\beta , -\alpha )$. Now we note that

$$\begin{aligned} (A,B)(\textbf{v} \otimes \textbf{u})= &   (A,B)(WW^{-1} \otimes I_n)(\textbf{v} \otimes \textbf{u})\\= &   (A,B)(W \otimes I_n)(W^{-1}\textbf{v} \otimes \textbf{u})\\  = &   L(A,B)(\overline{\textbf{v}} \otimes \textbf{u}) \end{aligned}$$

where we have defined $\overline{\textbf{v}}=W^{-1}\textbf{v}$. Thus, $\textbf{u}$ is also an eigenvector of *L*(*A*, *B*) and its associated eigenvalue is determined from $(\overline{\beta },-\overline{\alpha })=(\beta ,-\alpha )W^{-T}$. The statement follows after rewriting this relation and simplifying it thanks to (2.1). $\square$

Lemma 2.9 shows that there is a one-to-one relation between the eigenvalues of (*A*, *B*) and *L*(*A*, *B*) via the invertible matrix *W*. The name *linear fractional transformation* now takes on its full meaning since the transformed eigenvalues (in standard form) are given by

$$\begin{aligned} \overline{\lambda }=\frac{\overline{\alpha }}{\overline{\beta }}=\frac{w_{11}\alpha +w_{21}\beta }{w_{12}\alpha +w_{22}\beta }=\frac{w_{11}\lambda +w_{21}}{w_{12}\lambda +w_{22}}=f(\lambda ). \end{aligned}$$

On its interval of definition, one easily shows that $f(\lambda )$ is a strictly increasing (resp. strictly decreasing) function of $\lambda$ if $\det (W) > 0$ (resp. $\det (W) < 0$). Thus, the former preserves the eigenvalue numbering. Among the mass scaling strategies proposed in the literature, two of them are LFTs:

- *Uniform mass scaling* is an LFT with
  $$\begin{aligned} W= \begin{pmatrix} 1 &  0 \\ 0 &  \mu \end{pmatrix} \iff \overline{\lambda }=\frac{\lambda }{\mu }. \end{aligned}$$
- *Stiffness proportional* SMS [1, 23] in an LFT with
  $$\begin{aligned} W= \begin{pmatrix} 1 &  \mu \\ 0 &  1 \end{pmatrix} \iff \overline{\lambda }=\frac{\lambda }{\mu \lambda +1}. \end{aligned}$$

Uniform mass scaling simply scales all eigenvalues by $\mu > 0$ and therefore also affects the smallest ones. This impacts the accuracy of the dynamics, as testified by the numerical experiments in [26]. On the contrary, if the scaling parameter is wisely chosen, stiffness proportional mass scaling allows to selectively scale down the largest eigenvalues while nearly preserving the smallest ones. Olovsson et al. [1] recommend choosing $\mu =10^k/\lambda _n$ with $k \in \mathbb {N}$. If $\lambda _n/\lambda _1 \gg 10^{k}$, the smallest eigenvalues are barely affected while the largest ones are roughly scaled down by a factor of $10^k$. In practice, computing $\mu$ only requires a (coarse) estimation for $\lambda _n$. Unfortunately, the pleasing property of stiffness proportional SMS comes at the price of a non-diagonal scaled mass matrix $\overline{M}=M+\mu K$. In fact, if one uses this scaled mass matrix in an explicit scheme for linear elasto-dynamics, one might as well consider using an implicit unconditionally stable Newmark method from the very start, whose workload per time step is similar. The method is even less suited to nonlinear problems undergoing large rotations and deformations as the stiffness matrix changes during the course of the simulation and is typically only accessible through matrix–vector products. Stiffness proportional SMS would require reassembling the stiffness matrix at each time step, which is prohibitively expensive and is the main reason why, according to some authors [30, 42], the method has not been implemented in commercial software packages. Nevertheless, according to Askes et al. [62], stiffness proportional SMS is employed in other branches of mechanics, albeit under different names.

Instead of working with the original matrix pair (*A*, *B*), if *B* is invertible, one might consider the equivalent pair $(B^{-1}A,I)$, which shares the same eigenvalues and eigenvectors as (*A*, *B*). This reduces the generalized eigenvalue problem to standard form and enabled the authors in [24] to generalize the LFT to rational polynomials and even virtually any function. Given a matrix $A \in \mathbb {C}^{n \times n}$ and a function $f :\mathbb {C} \rightarrow \mathbb {C}$ well defined on the spectrum of *A*, it is well-known that if $(\lambda , \textbf{u})$ is an eigenpair of *A*, then $(f(\lambda ),\textbf{u})$ is an eigenpair of the matrix function *f*(*A*). Herein, *f*(*A*) is obtained from *f*(*t*) by simply substituting the indeterminate *t* with the matrix *A*, replacing 1 with the identity matrix and interpreting divisions as matrix inverses (if invertible). For further details, the interested reader may refer to [63, Chapter 1] for an introduction to matrix functions. In [24] the authors applied this general result to $M^{-1}K$ with a function

$$\begin{aligned} f(t) = \frac{t}{1+p(t)} \end{aligned}$$

where *p*(*t*) is a real coefficient polynomial of degree *d*. After rewriting $f(M^{-1}K)$ as a matrix pair, they considered using $(K,M(I + p(M^{-1}K)))$ with the second degree polynomial $p(t)=ct^2$ for some coefficient $c>0$. Although the method (refered to therein as *polynomial matrix* SMS) generalizes uniform and stiffness proportional SMS to arbitrary polynomials, it suffers from the same drawbacks as stiffness proportional SMS. Moreover, it explicitly features the inverse of *M* for any degree $d \ge 2$ and is therefore practically limited to diagonal (lumped) mass matrices. We are not aware of any further research alleviating this issue.

#### Global deflation

Instead of transforming the entire spectrum, it is also possible to modify only a few selected eigenvalues. In numerical linear algebra, this procedure is commonly known as *deflation*. Its origin dates back to the early work of Hotelling [22] and is well-known for standard eigenproblems. For generalized eigenproblems, the procedure is described e.g. in [60]. For $C^0$ finite element discretizations, González and Park [25] revisited this idea and improved the earlier work of Tkachuk and Bischoff [24] by proposing a fast solver for the scaled mass matrix based on the Woodbury matrix identity. While the method remains impractical for $C^0$ discretizations due to the sheer number of inaccurate frequencies, it becomes much more appealing for maximally smooth isogeometric discretizations featuring far fewer inaccurate frequencies [11, 13, 19]. It is briefly recalled here.

##### Definition 2.10

(Scaled matrix pair) Let $(A,B) \in \mathcal {S}_n \times \mathcal {S}_n^+$ and *f*, *g* be two functions defined on the spectrum of (*A*, *B*). The scaled matrix pair $(\overline{A},\overline{B})$ is defined as

$$\begin{aligned} \overline{A}&= A+Vf(D_2)V^T, \\ \overline{B}&= B+Vg(D_2)V^T, \end{aligned}$$

where $V=BU_2 \in \mathbb {R}^{n \times r}$, with $U_2=[\textbf{u}_{n-r+1}, \dots , \textbf{u}_n]$ the matrix formed by the last *r* *B*-orthonormal eigenvectors of (*A*, *B*) and $D_2={{\,\mathrm{\operatorname {diag}}\,}}(\lambda _{n-r+1}, \dots , \lambda _n) \in \mathbb {R}^{r \times r}$ the diagonal matrix formed by the last *r* eigenvalues with $r \ll n$.

##### Theorem 2.11

([19, Theorem 4.3]) Let $(A,B) \in \mathcal {S}_n \times \mathcal {S}_n^+$ and $(\overline{A},\overline{B})$ be the scaled matrix pair introduced in Definition 2.10. Then,

- The eigenvectors of (*A*, *B*) and $(\overline{A},\overline{B})$ are the same.
- The eigenvalues of $(\overline{A},\overline{B})$ are given by:
  $$\begin{aligned} \overline{\lambda }_{i_k}= {\left\{ \begin{array}{ll} \lambda _k &  \text { for } k=1,\dots ,n-r, \\ \frac{\lambda _k+f(\lambda _k)}{1+g(\lambda _k)} &  \text { for } k=n-r+1,\dots ,n. \end{array}\right. } \end{aligned}$$

In the context of mass scaling, one typically sets

2.2 $$\begin{aligned} f(\lambda )=0 \quad \text {and} \quad g(\lambda )=\frac{\lambda }{\lambda _{n-r}}-1, \end{aligned}$$

and the transformed eigenvalues are then given by

$$\begin{aligned} \overline{\lambda }_{k}= {\left\{ \begin{array}{ll} \lambda _k &  \text { for } k=1,\dots ,n-r, \\ \lambda _{n-r} &  \text { for } k=n-r+1,\dots ,n. \end{array}\right. } \end{aligned}$$

**Fig. 1:**
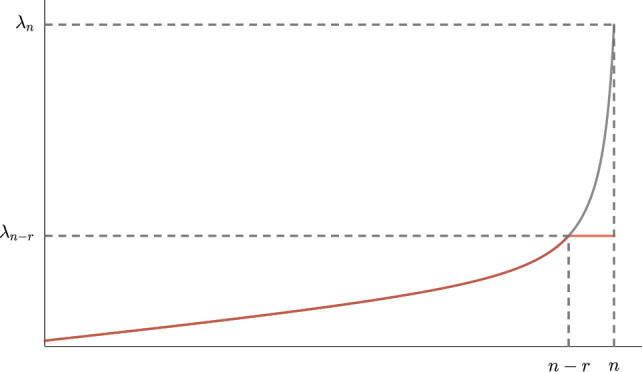
Truncation of the largest eigenvalues

The choices of *f* and *g* are not unique and one may equivalently choose

$$\begin{aligned} f(\lambda )=\lambda _{n-r}-\lambda \quad \text {and} \quad g(\lambda )=0, \end{aligned}$$

which would instead scale the stiffness matrix. Optionally, $\lambda _{n-r}$ can be substituted with a cutoff value, as suggested in [24, 25]. However, choosing $\lambda _{n-r}$ preserves the eigenvalue ordering and graphically shaves off the upper part of the spectrum (see Fig. 1). The increase of the critical time step for the central difference method is directly appreciated since

$$\begin{aligned} \frac{\overline{\Delta t}_c}{\Delta t_c}=\sqrt{\frac{\lambda _n}{\lambda _{n-r}}}. \end{aligned}$$

Although the scaled mass matrix is non-diagonal, [25, Equation (28)] provides an explicit expression for its inverse, based on the Woodbury matrix identity. Unfortunately, this strategy still requires computing some of the largest eigenpairs of a (generally very large) matrix pair, which is prohibitively expensive for $C^0$ finite element discretizations due to the “optical branches” of the spectrum contributing too many inaccurate high frequencies. The method becomes much more affordable and even realistic for maximally smooth isogeometric discretizations featuring far fewer inaccurate high frequencies [19, Theorems A.1 and A.2] whose separation from the rest of the spectrum is also a key advantage in accelerating eigensolvers based on Krylov subspaces. Alternatively, deflation strategies may rely on low-frequency modes combined with a projector in the orthogonal complement [25]. Although such methods do not offer any computational advantages in this setting, they form the cornerstone of inexact ad hoc deflation strategies reviewed in Sections 2.2.4 and 2.2.5

Theorem 2.11 shows that the eigenspaces of the original and scaled matrix pairs defined in Definition 2.10 are exactly the same. For more general mass scaling strategies, they are usually different although they might intersect. The next lemma recalls an elementary albeit important fact.

##### Lemma 2.12

If $(\lambda , \textbf{u})$ and $(\mu , \textbf{u})$ are eigenpairs of (*A*, *B*) and (*B*, *C*), respectively, then $(\lambda \mu , \textbf{u})$ is an eigenpair of (*A*, *C*).

##### Proof

If $(\lambda , \textbf{u})$ and $(\mu , \textbf{u})$ are eigenpairs of (*A*, *B*) and (*B*, *C*), respectively, then

$$\begin{aligned} A\textbf{u}=\lambda B \textbf{u} = \lambda \mu C \textbf{u} \end{aligned}$$

showing that $\lambda \mu$ is an eigenvalue of (*A*, *C*). $\square$

The previous result remains practically relevant in case the eigenspaces nearly intersect. This is also probably the main reason underpinning the success of mass lumping strategies, which tend to well approximate low-frequency eigenmodes. Note that if a vector $\textbf{u}$ is known to be an eigenvector of (*A*, *B*) and (*B*, *C*) with $A \in \mathcal {S}_n$ and $B, C \in \mathcal {S}_n^+$, then the corresponding eigenvalue of (*A*, *C*) may be directly computed as

$$\begin{aligned} \lambda (A,C)=\frac{R(\textbf{u})}{Q(\textbf{u})}, \end{aligned}$$

where

2.3 $$\begin{aligned} R(\textbf{x})=\frac{\textbf{x}^T A \textbf{x}}{\textbf{x}^T B \textbf{x}} \quad \text {and} \quad Q(\textbf{x})=\frac{\textbf{x}^T C \textbf{x}}{\textbf{x}^T B \textbf{x}} \end{aligned}$$

are the Rayleigh quotients.

### Local mass scaling

#### Preliminaries

Some of the global methods we have previously reviewed also have a local counterpart. The two approaches rarely coincide, expect for uniform or stiffness proportional SMS, provided the scaling parameter is chosen uniformly across elements. In general though, fully assembled system matrices do not immediately inherit local properties from element contributions. Nevertheless, there often exist very useful connections. We will first recall some of them and later analyze a few practical schemes proposed in the literature. Given the prominent nature of the following theorem, its proof is included for completeness.

##### Theorem 2.13

([4, Theorem 3.4]) Let $A \in \mathcal {S}_n$, $B,C \in \mathcal {S}_n^+$ and let all eigenvalues be numbered in ascending algebraic order. Then

2.4a $$\begin{aligned} \lambda _k(A,C)\lambda _1(C,B)\le &   \lambda _k(A,B) \le \lambda _k(A,C)\lambda _n(C,B)\qquad 1 \le k \le n, \nonumber \\ \end{aligned}$$

2.4b $$\begin{aligned} \lambda _1(A,C)\lambda _k(C,B)\le &   \lambda _k(A,B) \le \lambda _n(A,C)\lambda _k(C,B)\qquad 1 \le k \le n. \nonumber \\ \end{aligned}$$

##### Proof

Let $\textbf{u}_i$, $\textbf{v}_i$ and $\textbf{w}_i$ denote the eigenvectors associated to the eigenvalues $\lambda _i(A,B)$, $\lambda _i(A,C)$ and $\lambda _i(C,B)$, respectively. We define the subspaces

$$\begin{aligned} \mathcal {U}_k&= {{\,\mathrm{\operatorname {span}}\,}}\{\textbf{u}_1, \dots , \textbf{u}_k \},&\mathcal {U}'_k&= {{\,\mathrm{\operatorname {span}}\,}}\{\textbf{u}_k, \dots , \textbf{u}_n \}, \\ \mathcal {V}_k&= {{\,\mathrm{\operatorname {span}}\,}}\{\textbf{v}_1, \dots , \textbf{v}_k \},&\mathcal {V}'_k&= {{\,\mathrm{\operatorname {span}}\,}}\{\textbf{v}_k, \dots , \textbf{v}_n \}, \\ \mathcal {W}_k&= {{\,\mathrm{\operatorname {span}}\,}}\{\textbf{w}_1, \dots , \textbf{w}_k \},&\mathcal {W}'_k&= {{\,\mathrm{\operatorname {span}}\,}}\{\textbf{w}_k, \dots , \textbf{w}_n \}. \end{aligned}$$

Since $A \in \mathcal {S}_n$ and $B,C \in \mathcal {S}_n^+$, the generalized eigenvectors of all matrix pairs are linearly independent (see Lemma 2.4). Consequently, $\dim (\mathcal {U}_k)=\dim (\mathcal {V}_k)=\dim (\mathcal {W}_k)=k$ and $\dim (\mathcal {U}'_k)=\dim (\mathcal {V}'_k)=\dim (\mathcal {W}'_k)=n-k+1$. By the Courant-Fischer theorem (see for instance [64, Theorem 4.2.6])

$$\begin{aligned} \lambda _k(A,B)= &   \min _{\begin{array}{c} \mathcal {S} \subseteq \mathbb {R}^n \\ \dim (\mathcal {S})=k \end{array}} \max _{\begin{array}{c} \textbf{x} \in \mathcal {S} \\ \textbf{x} \ne \textbf{0} \end{array}} \frac{\textbf{x}^T A\textbf{x}}{\textbf{x}^T B \textbf{x}} = \\  &   \max _{\begin{array}{c} \mathcal {S} \subseteq \mathbb {R}^n \\ \dim (\mathcal {S})=n-k+1 \end{array}} \min _{\begin{array}{c} \textbf{x} \in \mathcal {S} \\ \textbf{x} \ne \textbf{0} \end{array}} \frac{\textbf{x}^T A\textbf{x}}{\textbf{x}^T B \textbf{x}} \qquad 1 \le k \le n. \end{aligned}$$

We already know that the minimum in the second expression is attained for $\mathcal {S}=\mathcal {U}_k$ and the maximum in the third expression is attained for $\mathcal {S}=\mathcal {U}'_k$. Moreover, we note that

$$\begin{aligned} \lambda _k(A,B)= &   \min _{\begin{array}{c} \mathcal {S} \subseteq \mathbb {R}^n \\ \dim (\mathcal {S})=k \end{array}} \max _{\begin{array}{c} \textbf{x} \in \mathcal {S} \\ \textbf{x} \ne \textbf{0} \end{array}} \frac{\textbf{x}^T A\textbf{x}}{\textbf{x}^T C \textbf{x}}\frac{\textbf{x}^T C\textbf{x}}{\textbf{x}^T B \textbf{x}}\\\le &   \max _{\begin{array}{c} \textbf{x} \in \mathcal {X}_k \\ \textbf{x} \ne \textbf{0} \end{array}} \frac{\textbf{x}^T A\textbf{x}}{\textbf{x}^T C \textbf{x}}\frac{\textbf{x}^T C\textbf{x}}{\textbf{x}^T B \textbf{x}}\\\le &   \max _{\begin{array}{c} \textbf{x} \in \mathcal {X}_k \\ \textbf{x} \ne \textbf{0} \end{array}} \frac{\textbf{x}^T A\textbf{x}}{\textbf{x}^T C \textbf{x}} \max _{\begin{array}{c} \textbf{x} \in \mathcal {X}_k \\ \textbf{x} \ne \textbf{0} \end{array}} \frac{\textbf{x}^T C\textbf{x}}{\textbf{x}^T B \textbf{x}}, \end{aligned}$$

and

$$\begin{aligned} \lambda _k(A,B)= &   \max _{\begin{array}{c} \mathcal {S} \subseteq \mathbb {R}^n \\ \dim (\mathcal {S})=n-k+1 \end{array}} \min _{\begin{array}{c} \textbf{x} \in \mathcal {S} \\ \textbf{x} \ne \textbf{0} \end{array}} \frac{\textbf{x}^T A\textbf{x}}{\textbf{x}^T C \textbf{x}}\frac{\textbf{x}^T C \textbf{x}}{\textbf{x}^T B \textbf{x}}\\\ge &   \min _{\begin{array}{c} \textbf{x} \in \mathcal {X}'_k \\ \textbf{x} \ne \textbf{0} \end{array}} \frac{\textbf{x}^T A\textbf{x}}{\textbf{x}^T C \textbf{x}}\frac{\textbf{x}^T C\textbf{x}}{\textbf{x}^T B \textbf{x}}\\\ge &   \min _{\begin{array}{c} \textbf{x} \in \mathcal {X}'_k \\ \textbf{x} \ne \textbf{0} \end{array}} \frac{\textbf{x}^T A\textbf{x}}{\textbf{x}^T C \textbf{x}} \min _{\begin{array}{c} \textbf{x} \in \mathcal {X}'_k \\ \textbf{x} \ne \textbf{0} \end{array}} \frac{\textbf{x}^T C\textbf{x}}{\textbf{x}^T B \textbf{x}}, \end{aligned}$$

for any given subspaces $\mathcal {X}_k \subseteq \mathbb {R}^n$ and $\mathcal {X}'_k \subseteq \mathbb {R}^n$ with $\dim (\mathcal {X}_k)=k$ and $\dim (\mathcal {X}'_k)=n-k+1$. The strategy now consists in cleverly choosing these subspaces. In particular, for the upper bound, we obtain

$$\begin{aligned} \lambda _k(A,B)&\le \lambda _k(A,C) \lambda _n(C,B) \qquad \text {for } \mathcal {X}_k=\mathcal {V}_k, \\ \lambda _k(A,B)&\le \lambda _n(A,C) \lambda _k(C,B) \qquad \text {for } \mathcal {X}_k=\mathcal {W}_k, \end{aligned}$$

and for the lower bound

$$\begin{aligned} \lambda _k(A,B)&\ge \lambda _k(A,C) \lambda _1(C,B) \qquad \text {for } \mathcal {X}'_k=\mathcal {V}'_k, \\ \lambda _k(A,B)&\ge \lambda _1(A,C) \lambda _k(C,B) \qquad \text {for } \mathcal {X}'_k=\mathcal {W}'_k. \end{aligned}$$

$$\square $$

##### Remark 2.14

One may derive a slight improvement of Theorem 2.13. By carefully inspecting the proof steps, the following sharper bounds hold:

$$\begin{aligned} \lambda _k(A,C)\min _{\begin{array}{c} \textbf{x} \in \mathcal {V}'_k \\ \textbf{x} \ne \textbf{0} \end{array}} Q(\textbf{x}) \le \lambda _k(A,B) \le \lambda _k(A,C) \max _{\begin{array}{c} \textbf{x} \in \mathcal {V}_k \\ \textbf{x} \ne \textbf{0} \end{array}} Q(\textbf{x}) \qquad 1 \le k \le n \end{aligned}$$

where $Q(\textbf{x})$ is the Rayleigh quotient defined in eq. (2.3). A direct application of the previous inequality, with $A=K$, $B=M$ and $C=\overline{M}$, yields

2.5 $$\begin{aligned} \min _{\begin{array}{c} \textbf{x} \in \mathcal {V}'_k \\ \textbf{x} \ne \textbf{0} \end{array}} Q(\textbf{x}) \le \frac{\lambda _k(K,M)}{\lambda _k(K,\overline{M})} \le \max _{\begin{array}{c} \textbf{x} \in \mathcal {V}_k \\ \textbf{x} \ne \textbf{0} \end{array}} Q(\textbf{x}) \qquad 1 \le k \le n. \end{aligned}$$

Note that $\mathcal {V}_k$ and $\mathcal {V}'_k$ are eigenspaces for the perturbed matrix pair $(K,\overline{M})$. Thus, these bounds are analogous to a posteriori error estimates and underpin the selective nature of mass scaling techniques. However, the lower and upper bounds are difficult to compute and we will often prefer the coarser but more explicit bound of Theorem 2.13, inequalities (2.4a)

2.6 $$\begin{aligned} \lambda _1(\overline{M},M) \le \frac{\lambda _k(K,M)}{\lambda _k(K,\overline{M})} \le \lambda _n(\overline{M},M) \qquad 1 \le k \le n. \end{aligned}$$

This inequality will play a central role for deriving time step estimates.

As we have discussed, bounds on the condition number of the scaled mass matrix are also desirable if linear systems with the latter are solved iteratively. We recall that the spectral condition number of a symmetric positive definite matrix *A* is defined as $\kappa (A)=\lambda _n(A)/\lambda _1(A)$. Analogously, we define the condition number of a pair of symmetric positive definite matrices (*A*, *B*) as $\kappa (A,B)=\lambda _n(A,B)/\lambda _1(A,B)$. The next corollary provides a useful inequality relating the two.

##### Corollary 2.15

Let $A,B \in \mathcal {S}_n^+$ and let $\kappa$ denote the spectral condition number. Then

$$\begin{aligned} \frac{\kappa (A)}{\kappa (B)} \le \kappa (A,B). \end{aligned}$$

##### Proof

Applying Theorem 2.13, inequalities (2.4a) with $C=I$ yields

$$\begin{aligned} \lambda _1(A,B) \le \frac{\lambda _1(A)}{\lambda _1(B)} \quad \text {and} \quad \frac{\lambda _n(A)}{\lambda _n(B)} \le \lambda _n(A,B). \end{aligned}$$

Combining the two previous inequalities concludes the proof. $\square$

So far, all inequalities pertain to the global system matrices. Yet, the properties of finite element matrices are usually related to those of the element matrices from which they are formed. The two are connected through the assembly procedure, which we describe next. To simplify the presentation, we assume that the mesh is made up of a single type of element such that all element matrices have the same size *m*. Then, given a set of element matrices $\{A_e\}_{e=1}^N \subseteq \mathcal {S}_m$, where *N* is the number of elements, the global matrix $A \in \mathbb {R}^{n \times n}$ can be expressed as

2.7 $$\begin{aligned} A=\sum _{e=1}^N L_e^TA_eL_e, \end{aligned}$$

where $L_e^T = [\textbf{e}_{i_1},\dots ,\textbf{e}_{i_m}] \in \mathbb {R}^{n \times m}$ contains a subset of the columns of the identity matrix $I_n$ with indices $i_k$ for $k=1,\dots ,m$ depending on the connectivity of the element. Denoting $\textsf{L}^T=[L_1^T, \dots , L_N^T] \in \mathbb {R}^{n \times mN}$ and $\textsf{A}={{\,\mathrm{\operatorname {diag}}\,}}(A_1, \dots , A_N) \in \mathbb {R}^{mN \times mN}$, eq. (2.7) is compactly written as $A=\textsf{L}^T\textsf{A}~\textsf{L}$. With this expression at hand, we now summarize some of the main results of [65, 66]. The proofs are included and will be useful later in the analysis.

##### Lemma 2.16

([65, Theorem 1]) Given a finite element matrix $A=\textsf{L}^T\textsf{A}\textsf{L}$. Then

$$\begin{aligned} \max _e \lambda _m(A_e)&\le \lambda _n(A) \le p_{\max } \max _e \lambda _m(A_e), \\ \min _e \lambda _1(A_e)&\le \lambda _1(A), \end{aligned}$$

where $p_{\max } \in \mathbb {N}^*$ is the maximum number of elements to which a node is connected.

##### Proof

Given $$\textbf{x} \in \mathbb {R}^n$$ with $$\Vert \textbf{x}\Vert _2=1$$,

$$\begin{aligned} \lambda _{1}(\textsf{A})\Vert \textsf{L}\textbf{x}\Vert _2^2 \le \textbf{x}^T A \textbf{x} =\textbf{x}^T\textsf{L}^T\textsf{A}\textsf{L}\textbf{x} \le \lambda _{mN}(\textsf{A})\Vert \textsf{L}\textbf{x}\Vert _2^2. \end{aligned}$$

Since $\textsf{A}$ is block-diagonal, $\lambda _{1}(\textsf{A})=\min _e \lambda _1(A_e)$ and $\lambda _{mN}(\textsf{A})=\max _e \lambda _m(A_e)$. Moreover,

$$\begin{aligned} \Vert \textsf{L}\textbf{x}\Vert _2^2=\textbf{x}^T\textsf{L}^T\textsf{L}\textbf{x}=\sum _{e=1}^N \textbf{x}^TL_e^TL_e\textbf{x}=\sum _{e=1}^N \Vert \textbf{x}_e\Vert _2^2 = \sum _{i=1}^n p_i x_i^2 \end{aligned}$$

where $\textbf{x}_e=L_e\textbf{x}$ is a subvector of $\textbf{x}$ and $p_i$ is the number of elements connected to node *i*. Since $1 \le p_i \le p_{\max }$ for all $i=1,\dots ,n$ and $\Vert \textbf{x}\Vert _2=1$, we immediately deduce that $1 \le \Vert \textsf{L}\textbf{x}\Vert _2^2 \le p_{\max }$. Combining these results, we obtain

$$\begin{aligned} \min _e \lambda _1(A_e) \le \textbf{x}^T A\textbf{x} \le p_{\max } \max _e \lambda _m(A_e), \end{aligned}$$

which yields an upper bound on $\lambda _n(A)$ and a lower bound on $\lambda _1(A)$. The lower bound on $\lambda _n(A)$ is deduced for a specific choice of vector. Namely, we choose $\textbf{x}$ to have zero components everywhere except for the subvector $\textbf{x}_e$ corresponding to the element with largest eigenvalue, which we call *l*. Then, $\Vert \textbf{x}\Vert _2=\Vert \textbf{x}_l\Vert _2=1$ and by choosing $\textbf{x}_l$ to be the eigenvector associated to the largest eigenvalue of $A_l$, we finally obtain

$$\begin{aligned} \lambda _n(A) \ge \textbf{x}^T A \textbf{x} = \sum _{e=1}^N \textbf{x}_e^T A_e \textbf{x}_e \ge \textbf{x}_l^T A_l \textbf{x}_l= \max _e \lambda _m(A_e), \end{aligned}$$

where the second inequality follows from the positive semi-definiteness of $A_e$ for $e=1,\dots ,N$. $\square$

The previous lemma can be used to compute an upper bound on the condition number of the mass matrix as [65, Corollary 1]

2.8 $$\begin{aligned} 1 \le \kappa (M) \le p_{\max } \frac{\max _e \lambda _m(M_e)}{\min _e \lambda _1(M_e)}. \end{aligned}$$

For immersed finite element discretizations, the condition number may become unbounded as $\lambda _1(M_e)$ goes to zero for arbitrarily small cut elements.

The next lemma, attributed to Irons and Treharne [67], has already appeared numerous times in the finite element literature (see e.g. [6, 66]). Its short and elegant proof, due to Wathen [66], is included for completeness.

##### Theorem 2.17

([67]) Let $\{A_e\}_{e=1}^N \subseteq \mathcal {S}_m$ and $\{B_e\}_{e=1}^N \subseteq \mathcal {S}_m^+$ be sets of element matrices for global finite element matrices *A* and *B*, respectively, and *N* be the number of elements. Then

$$\begin{aligned}  &   \min _e \lambda _1(A_e,B_e) \le \lambda _1(A,B), \qquad \\  &   \lambda _n(A,B) \le \max _e \lambda _m(A_e,B_e). \end{aligned}$$

##### Proof

$$\begin{aligned} \lambda _1(A,B)= &   \min _{\begin{array}{c} \textbf{x} \in \mathbb {R}^n \\ \textbf{x} \ne \textbf{0} \end{array}} \frac{\textbf{x}^T \textsf{L}^T\textsf{A}\textsf{L}\textbf{x}}{\textbf{x}^T \textsf{L}^T\textsf{B}\textsf{L} \textbf{x}}=\min _{\begin{array}{c} \textbf{y} \in \mathcal {Y} \\ \textbf{y} \ne \textbf{0} \end{array}} \frac{\textbf{y}^T \textsf{A} \textbf{y}}{\textbf{y}^T \textsf{B} \textbf{y}} \\\ge &   \min _{\begin{array}{c} \textbf{y} \in \mathbb {R}^{mN} \\ \textbf{y} \ne \textbf{0} \end{array}} \frac{\textbf{y}^T \textsf{A} \textbf{y}}{\textbf{y}^T \textsf{B} \textbf{y}}=\min _e \lambda _1(A_e,B_e), \end{aligned}$$

where $\mathcal {Y} \subseteq \mathbb {R}^{mN}$ is the space spanned by the columns of $\textsf{L}$. Similarly,

$$\begin{aligned} \lambda _n(A,B)= &   \max _{\begin{array}{c} \textbf{x} \in \mathbb {R}^n \\ \textbf{x} \ne \textbf{0} \end{array}} \frac{\textbf{x}^T \textsf{L}^T\textsf{A}\textsf{L}\textbf{x}}{\textbf{x}^T \textsf{L}^T\textsf{B}\textsf{L} \textbf{x}}=\max _{\begin{array}{c} \textbf{y} \in \mathcal {Y} \\ \textbf{y} \ne \textbf{0} \end{array}} \frac{\textbf{y}^T \textsf{A} \textbf{y}}{\textbf{y}^T \textsf{B} \textbf{y}} \\\le &   \max _{\begin{array}{c} \textbf{y} \in \mathbb {R}^{mN} \\ \textbf{y} \ne \textbf{0} \end{array}} \frac{\textbf{y}^T \textsf{A} \textbf{y}}{\textbf{y}^T \textsf{B} \textbf{y}}=\max _e \lambda _m(A_e,B_e). \end{aligned}$$

$$\square $$

The upper bound of Theorem 2.17 is usually tight [68] and translates into a relatively large and conservative step size estimate for applications in explicit dynamics. However, neither Theorem 2.17 nor Lemma 2.16 are applicable for bounding the eigenvalues of *CK*, where *C* is a reciprocal mass matrix (i.e. a sparse approximate inverse of the mass matrix) [42, 43]. Indeed, *CK* does not have the form described in eq. (2.7) and is generally not even symmetric. Instead, upper bounds based on the classical Gershgorin circle theorem [50] or its generalization given by Ostrowski’s bound [52] have been suggested. In [27], the authors have noted that the former produced large overestimates for the element largest eigenfrequency when using $M^{-1}K$. This is not surprising given that pre-multiplication of *K* with the diagonal matrix $M^{-1}$ will only rescale its rows. Substituting $M^{-1}K$ with the similar (and symmetric) matrix $M^{-1/2}KM^{-1/2}$ sometimes produces sharper bounds [51]. Combining the Gershgorin theorem with a diagonal similarity transformation might also improve the bounds [64, Corollary 6.1.6], particularly for beam, plate or shell problems, when the matrix entries have different physical units [52].

In contrast, several authors in the engineering community have undertaken the task of deriving analytical or heuristic estimates for the largest element frequencies (see e.g. [27, 28, 47, 48]). However, these estimates are not necessarily conservative and rarely account for common changes to element formulations due, for instance, to reduced integration, locking-free formulations or the addition of penalty terms [40, 69]. Nevertheless, they may help identify critical elements [47], before using refined estimates.

As a matter of fact, the sharpness of the upper bound of Theorem 2.17 also highlights how individual elements might negatively impact the critical time step and is an incentive for modifying the element formulations prior to assembly. This reasoning drove early developments of local mass scaling techniques [26]. We are now ready to analyze some of the practical schemes proposed in the literature.

#### Conventional mass scaling

Conventional mass scaling only scales the diagonal entries of local (lumped) mass matrices. Without loss of generality, we may assume only the first *r* entries are scaled by a factor $\alpha >1$ such that if $M_e={{\,\mathrm{\operatorname {diag}}\,}}(d_1,\dots ,d_m)$, then $\overline{M}_e = {{\,\mathrm{\operatorname {diag}}\,}}(\alpha d_1, \dots ,\alpha d_r, d_{r+1},\dots ,d_m)$. In this case, the following corollary holds.

##### Corollary 2.18

For conventional mass scaling with a uniform scaling parameter $\alpha >1$ the global eigenfrequencies $\overline{\omega }_i$ and $\omega _i$ of the scaled and unscaled system, respectively, satisfy

$$\begin{aligned} 1 \le \frac{\omega _i}{\overline{\omega }_i} \le \sqrt{\alpha }. \end{aligned}$$

##### Proof

Combining Theorems 2.13 and 2.17

$$\begin{aligned} \min _e \lambda _1(\overline{M}_e, M_e)\le &   \lambda _1(\overline{M},M) \le \frac{\lambda _i(K,M)}{\lambda _i(K, \overline{M})} \\\le &   \lambda _n(\overline{M},M) \le \max _e \lambda _m(\overline{M}_e, M_e). \end{aligned}$$

Since only selected entries of the element lumped mass matrices $M_e$ are scaled by $\alpha >1$, $\lambda _1(\overline{M}_e, M_e)=1$ and $\lambda _m(\overline{M}_e, M_e)=\alpha$. The result then immediately follows after taking the square root. $\square$

Corollary 2.18 neither depends on the nature of the scaled degrees of freedom nor on the original physical model. Thus, it applies both to rotational mass scaling [20, 21] and to the more recent intrinsically selective mass scaling technique (ISMS) [29, 31]. While the sharpness of the bounds certainly depends on these factors, the numerical experiments reported in [31] for ISMS reveal that the upper bound is tight if $\alpha$ is not too large.

#### Local deflation

Several authors [25, 36] have suggested applying locally the eigenvalue deflation techniques reviewed in Section  2.1.3. Following the construction mentioned in [25, 37], all local mass matrices $\{M_e\}_{e=1}^N$ are scaled according to Definition 2.10 with $f(\lambda )=0$ and $g(\lambda )=\alpha$ and their contributions are assembled in the usual manner. For simplicity, we assume a uniform rank $0<r<m$ and cutoff value $\alpha >0$ for all elements. For this construction, the following corollary holds.

##### Corollary 2.19

If all element matrices are scaled following Definition 2.10 with $f(\lambda )=0$ and $g(\lambda )=\alpha$ for a uniform cutoff value of $\alpha$, then, the global eigenfrequencies $\overline{\omega }_i$ and $\omega _i$ of the scaled and unscaled system, respectively, satisfy

$$\begin{aligned} 1 \le \frac{\omega _i}{\overline{\omega }_i} \le \sqrt{1+\alpha }. \end{aligned}$$

##### Proof

The proof strategy is exactly the same as in Corollary 2.18 and only requires computing the eigenvalues of $(\overline{M}_e, M_e)$. Applying Theorem 2.11 locally (with $A=B=M_e$, $f(\lambda )=\alpha$ and $g(\lambda )=0$), we deduce that the eigenvalues of $(\overline{M}_e, M_e)$ are

$$\begin{aligned} \lambda _k(\overline{M}_e, M_e)= {\left\{ \begin{array}{ll} 1 &  \text { for } k=1,\dots ,m-r, \\ 1+\alpha &  \text { for } k=m-r+1,\dots ,m. \end{array}\right. } \end{aligned}$$

The result then immediately follows after taking the square root. $\square$

Corollaries 2.18 and 2.19 are strikingly similar and may achieve a similar increase of the critical time step. However, local deflation strategies may deliver far greater accuracy by specifically targeting restrictive modes that contribute very little to the solution. Selecting the right modes is strongly problem-dependent. For instance, for thin-walled structures, they are usually thickness stretch modes [27, 28, 35] while for nearly incompressible materials, they are volumetric modes [36]. However, if $\alpha$ becomes too large, other deformation modes may limit the critical time step and, as shown experimentally in [25, 37], the method then loses significant accuracy by introducing high-frequency modes in the low-frequency spectrum. This phenomenon is less likely if the eigenvalue numbering is preserved. Thus, the authors in [24, 25] directly applied a local counterpart of the global deflation technique (with the same functions *f* and *g* as globally) and a uniform rank value *r*. A similar technique was applied patchwise in [19] for isogeometric discretizations. Corollary 2.20 below provides the bounds in this case.

##### Corollary 2.20

If all element matrices are scaled following Definition 2.10 with the functions *f* and *g* defined in eq. (2.2), then, the global eigenfrequencies $\overline{\omega }_i$ and $\omega _i$ of the scaled and unscaled system, respectively, satisfy

$$\begin{aligned} 1 \le \frac{\omega _i}{\overline{\omega }_i} \le \max _e \frac{\omega _{m,e}}{\omega _{m-r,e}}, \end{aligned}$$

where $\omega _{j,e}=\sqrt{\lambda _j(K_e,M_e)}$ are the eigenfrequencies of $(K_e,M_e)$.

##### Proof

The proof merely requires adapting the choices of *f* and *g* in Corollary 2.19. $\square$

##### Remark 2.21

If the central difference method is used, Corollary 2.20 may be formulated as

$$\begin{aligned} 1 \le \frac{\overline{\Delta t}_c}{\Delta t_c} \le \max _e \frac{\overline{\Delta t}_{c,e}}{\Delta t_{c,e}}, \end{aligned}$$

where $\overline{\Delta t}_{c,e}$ and $\Delta t_{c,e}$ are the critical time steps for the locally scaled and unscaled systems, respectively.

The benefits of local deflation techniques are not always clear. On the one hand, their setup is cheaper than their global counterpart. On the other hand, they might affect the lower frequencies and be less effective at removing the larger ones. Moreover, the Woodbury identity no longer applies to locally deflated systems and solving linear systems with the locally scaled mass matrix is not straightforward. This was the main reason in [19] for locally scaling the stiffness matrices instead.

Element modifications have also been suggested for improving the conditioning of system matrices and the stability of immersed methods. In [38], the element mass (and stiffness) matrices of cut elements are modified by stabilizing near zero eigenvalues. Those techniques simply consist in locally modifying the spectrum of element matrices by adding a stabilization parameter to near zero eigenvalues. Assuming that the *r* smallest eigenvalues of the element mass matrix for a cut element *c* are stabilized with a parameter $\epsilon$ and

$$\begin{aligned} \lambda _1(M_c)+\epsilon\le &   \dots \le \lambda _r(M_c) + \epsilon \\\le &   \lambda _{r+1}(M_c) \le \dots \le \lambda _m(M_c), \end{aligned}$$

then, if one reasonably assumes that $\min _e \lambda _1(\overline{M}_e)$ is attained for the cut element, the improvement of the condition number of the assembled mass matrix is readily appreciated from eq. (2.8):

$$\begin{aligned} \kappa (\overline{M}) \le \frac{p_{\max }}{\epsilon } \max _e \lambda _m(M_e). \end{aligned}$$

Unfortunately, local deflation strategies require explicit knowledge of the mode shapes, which is not convenient when the stiffness matrix changes during the course of the simulation. Some alternatives are analyzed next.

#### Method of [Olovsson et al., 2005]

In the early 2000s, Olovsson et al. [1] proposed an ad hoc local mass scaling strategy specifically designed for linear hexahedral elements (and later generalized to other elements). For problems in elasticity, the element lumped mass and scaling matrices are defined as

$$\begin{aligned} M_e=I_3 \otimes \textrm{M}_e \quad \text {and} \quad E_e=I_3 \otimes \textrm{E}_e, \end{aligned}$$

where $\textrm{M}_e=\frac{m_e}{8}I_8$ with $m_e$ the mass of element *e* and

$$\begin{aligned} \textrm{E}_e=\frac{\beta m_e}{56} \begin{pmatrix} 7 &  -1 &  \cdots &  -1 \\ -1 &  7 &  &  \vdots \\ \vdots &  &  \ddots &  \\ -1 &  \cdots &  &  7 \end{pmatrix} \end{aligned}$$

where $\beta \ge 0$ is a scaling parameter. In principle, $\beta$ may depend on the element but we will not consider such cases here. We immediately notice that $\textrm{E}_e$ is symmetric positive semidefinite with zero row-sum. Indeed, by the Gershgorin circle theorem [50, Theorem 1.1], its (real) eigenvalues lie in the interval $[0, \ \frac{\beta m_e}{4}]$. The positive semi-definiteness carries over to the assembled matrix *E* and Lemma 2.5 guarantees a decrease of the generalized eigenvalues of the scaled matrix pair. Note that $\textrm{E}_e$ may be expressed as^1^

2.9 $$\begin{aligned} \textrm{E}_e=\frac{\beta m_e}{7}(I_8-\textbf{u}\textbf{u}^T) \end{aligned}$$

where $\textbf{u}=\frac{\textbf{e}}{\Vert \textbf{e}\Vert _2}$ is the normalized vector of all ones. The analogy with local deflation techniques is now evident since eq. (2.9) indicates that, up to a multiplicative constant, $\textrm{E}_e$ is the orthogonal projector into the orthogonal complement of rigid body translations ${{\,\mathrm{\operatorname {span}}\,}}\{\textbf{u}\}^\perp$ [32] and $\textbf{u} \in \ker (\textrm{E}_e)$. In the engineering community, this last property is known as the conservation of linear momentum (or translational inertia) [42]. Since $M_e$ is a scaled identity matrix (i.e. a scalar matrix), the generalized eigenvectors of $(K_e,M_e)$ are merely eigenvectors of $K_e$. Moreover, since $K_e$ is symmetric, its eigenvectors are orthogonal and from eq. (1.1), one may easily show that

$$\begin{aligned} \frac{\overline{\Delta t}_{c,e}}{\Delta t_{c,e}} = \sqrt{1+\frac{8}{7}\beta }. \end{aligned}$$

In fact, as we will prove below, the right-hand side is also an upper bound on $\frac{\overline{\Delta t}_c}{\Delta t_c}$ and favorably compares with the $\sqrt{1+\beta }$ factor increase reported by Olovsson et al. [45].^2^ Unfortunately, the authors also reported an increase of the condition number of $\overline{M}$ by roughly a factor $1+2\beta$. We will now prove these observations and even refine them.

##### Corollary 2.22

(Mass scaling of [Olovsson et al., 2005]) For the mass scaling method of Olovsson et al. [1], the following inequalities hold:

$$\begin{aligned} 1 \le \frac{\omega _i}{\overline{\omega }_i} \le \sqrt{1+\frac{8}{7}\beta }, \qquad \frac{\kappa (\overline{M})}{\kappa (M)} \le 1+\frac{8}{7}\beta . \end{aligned}$$

##### Proof

We first prove the inequalities on the ratio of eigenfrequencies. Once again, by combining Theorems 2.13 and 2.17,

2.10 $$\begin{aligned} \min _e \lambda _1(\overline{M}_e, M_e)\le &   \lambda _1(\overline{M},M) \le \frac{\lambda _i(K,M)}{\lambda _i(K, \overline{M})} \le \lambda _n(\overline{M},M) \nonumber \\  &   \quad \le \max _e \lambda _m(\overline{M}_e, M_e). \end{aligned}$$

Moreover, since the Kronecker product only increases the multiplicity of the eigenvalues of $(\overline{\textrm{M}}_e, \textrm{M}_e)$,

$$\begin{aligned} \Lambda (\overline{M}_e, M_e)=\Lambda (\overline{\textrm{M}}_e, \textrm{M}_e). \end{aligned}$$

Additionally, due to the simple structure of $\textrm{M}_e$, the eigenvalues of $(\overline{\textrm{M}}_e, \textrm{M}_e)$ are known explicitly. Denoting $\gamma _e=\frac{m_e}{56}$, we obtain $\textrm{M}_e=7\gamma _e I_8$ and recalling eq. (2.9), $\textrm{E}_e=\beta \gamma _e(8I_8-\textbf{e}\textbf{e}^T)$, which is the sum of a scaled identity and rank-1 matrix. Consequently, the scaled mass matrix is given by

$$\begin{aligned} \overline{\textrm{M}}_e=\textrm{M}_e+\textrm{E}_e=\gamma _e((7+8\beta )I_8-\beta \textbf{e}\textbf{e}^T). \end{aligned}$$

Thus,

$$\begin{aligned} \Lambda (\overline{\textrm{M}}_e, \textrm{M}_e)= &   \Lambda (\gamma _e((7+8\beta )I_8-\beta \textbf{e}\textbf{e}^T), 7\gamma _e I_8)\\= &   \Lambda ((1+\frac{8}{7}\beta ) I_8-\frac{\beta }{7}\textbf{e}\textbf{e}^T). \end{aligned}$$

The eigenvalues of this last matrix are known explicitly. Indeed, since $\lambda _8(\textbf{e}\textbf{e}^T)=\Vert \textbf{e}\Vert _2^2=8$ and $\lambda _k(\textbf{e}\textbf{e}^T)=0$ for $k=1,\dots ,7$, we deduce that

2.11 $$\begin{aligned} \lambda _k(\overline{\textrm{M}}_e, \textrm{M}_e)= {\left\{ \begin{array}{ll} 1 &  \text { for } k=1, \\ 1+\frac{8}{7}\beta &  \text { for } k=2,\dots ,8. \end{array}\right. } \end{aligned}$$

The result for the eigenfrequency ratio then immediately follows by taking the square root in (2.10).

For the upper bound on the condition number, we invoke Corollary 2.15,

2.12 $$\begin{aligned}  &   \frac{\kappa (\overline{M})}{\kappa (M)} \le \kappa (\overline{M},M)=\frac{\lambda _n(\overline{M},M)}{\lambda _1(\overline{M},M)} \le \frac{\max _e \lambda _m(\overline{M}_e, M_e)}{\min _e \lambda _1(\overline{M}_e, M_e)}\nonumber \\  &   =\frac{\max _e \lambda _8(\overline{\textrm{M}}_e, \textrm{M}_e)}{\min _e \lambda _1(\overline{\textrm{M}}_e, \textrm{M}_e)}, \end{aligned}$$

where the second inequality follows from Theorem 2.17 and the last equality follows from eigenvalue multiplicity. Finally, recalling (2.11), the result follows. $\square$

##### Remark 2.23

Interestingly, none of the bounds depend on $m_e$, which bears the element dependency. A more explicit upper bound on the condition number of $\overline{M}$ can be derived from Lemma 2.16 and eq. (2.8):

2.13 $$\begin{aligned} \kappa (\overline{M}) \le p_{\max }(1+\frac{8}{7}\beta ) \frac{\max _e m_e}{\min _e m_e}. \end{aligned}$$

One can easily see that for a sufficiently refined uniform mesh of linear hexahedral elements with uniform density, the condition number of *M* is $p_{\max }$. Indeed, all element lumped mass matrices are given by $M_e=\alpha I$, where $\alpha$ is a constant independent of the element and is contributed exactly once for a corner degree of freedom while it is contributed $p_{\max }$ times for an interior degree of freedom. In such cases, the bounds of Corollary 2.22 and eq. (2.13) coincide. Moreover, for the central difference method, Corollary 2.22 also immediately yields a bound on the ratio of critical time steps:

$$\begin{aligned} 1 \le \frac{\overline{\Delta t}_c}{\Delta t_c} = \frac{\omega _n}{\overline{\omega }_n} \le \sqrt{1+\frac{8}{7}\beta }. \end{aligned}$$

In particular, we notice that the upper bound is very close to the estimate of $\sqrt{1+\beta }$ given in [45]. Furthermore, the proof strategy of Corollary 2.22 can be easily adapted to other elements.

#### Method of [Hoffmann et al., 2023]

A rather straightforward generalization of the method of Olovsson et al. consists in defining

2.14 $$\begin{aligned} \textrm{E}_e=\frac{\beta m_e}{7}(I_8-UU^T) \end{aligned}$$

where $U \in \mathbb {R}^{8 \times r}$ is a matrix whose columns form an orthonormal basis for the low-frequency mode shapes, including rigid body translations and rotations. In engineering terms, this mass scaling strategy preserves both linear and angular momentum (or translational and rotational inertia). By including more of the low-frequency content locally, one may hope for greater accuracy globally. Recently, Hoffmann et al. [35] suggested an improvement of the method of Olovsson et al. somewhat mimicking this idea and claiming similar increases on the critical time step while achieving significantly better accuracy for the smallest eigenvalues. The element scaling matrix is defined as $E_e= I_3 \otimes \textrm{E}_e$, where

$$\begin{aligned} \textrm{E}_e = \frac{\beta \widetilde{\gamma }_e}{4}(A \otimes G) \end{aligned}$$

with

$$\begin{aligned} A = \begin{pmatrix} 1 &  -1 \\ -1 &  1 \end{pmatrix}, \quad \text {and} \quad G = \begin{pmatrix} 4 &  2 &  1 &  2 \\ 2 &  4 &  2 &  1 \\ 1 &  2 &  4 &  2 \\ 2 &  1 &  2 &  4 \end{pmatrix}. \end{aligned}$$

The factor $\widetilde{\gamma }_e$ depends on the element geometry and material but is not explicitly defined by the authors in [35]. However, since their method builds on the work of Olovsson et al., we assume that $\tilde{\gamma }_e=7\gamma _e=\frac{m_e}{8}$ such that $\beta =1$ doubles the diagonal entries of $M_e$, similarly to the method of Olovsson et al.

##### Corollary 2.24

(Mass scaling of [Hoffmann et al., 2023]) For the mass scaling method of Hoffmann et al. [35], the following inequalities hold:

$$\begin{aligned} 1 \le \frac{\omega _i}{\overline{\omega }_i} \le \sqrt{1+\frac{9}{2}\beta }, \qquad \frac{\kappa (\overline{M})}{\kappa (M)} \le 1+\frac{9}{2}\beta . \end{aligned}$$

##### Proof

The proof arguments follow exactly the same lines as in Corollary 2.22. Only the definition of the scaling matrix changes and so do the eigenvalues of $(\overline{\textrm{M}}_e, \textrm{M}_e)$. Yet, computing them is again a simple exercise:

$$\begin{aligned} \Lambda (\overline{\textrm{M}}_e, \textrm{M}_e)= &   \Lambda (7\gamma _e ( I_8 + \frac{\beta }{4}(A \otimes G)), 7\gamma _e I_8) \\  = &   1+\frac{\beta }{4}\Lambda (A)\Lambda (G), \end{aligned}$$

where the multiplication of two sets is the set containing all elementwise multiplications of any two elements from the sets. The spectrum of *A* and *G* (with multiplicities) is

$$\begin{aligned} \Lambda (A) = \{0,2\}, \quad \text {and} \quad \Lambda (G) = \{1,3,3,9\}. \end{aligned}$$

Consequently,

2.15 $$\begin{aligned} \lambda _k(\overline{\textrm{M}}_e, \textrm{M}_e)= {\left\{ \begin{array}{ll} 1 &  \text { for } k=1,2,3,4, \\ 1+\frac{\beta }{2} &  \text { for } k=5, \\ 1+\frac{3\beta }{2} &  \text { for } k=6,7, \\ 1+\frac{9\beta }{2} &  \text { for } k=8. \end{array}\right. } \end{aligned}$$

The results then follow from direct substitution in (2.10) and (2.12). $\square$

In particular, Corollaries 2.22 and 2.24 suggest that increasing the critical time step and retaining a moderate condition number for the scaled mass matrix are conflicting objectives. This has already been observed numerically by several authors and will be confirmed in the next section.

## Numerical validation

This section provides a few numerical experiments supporting our theoretical findings. Our experiments specifically focus on local methods, when the transformed eigenvalues of the global system are unknown. We refer to the original articles cited herein for further experiments assessing the accuracy, especially for transient problems. The values reported for the critical time step are the ones for the central difference method eq. (1.1), ubiquitous in explicit dynamics. All experiments were done with an in-house finite element code while the meshes were generated with GMSH [70].

**Fig. 2:**
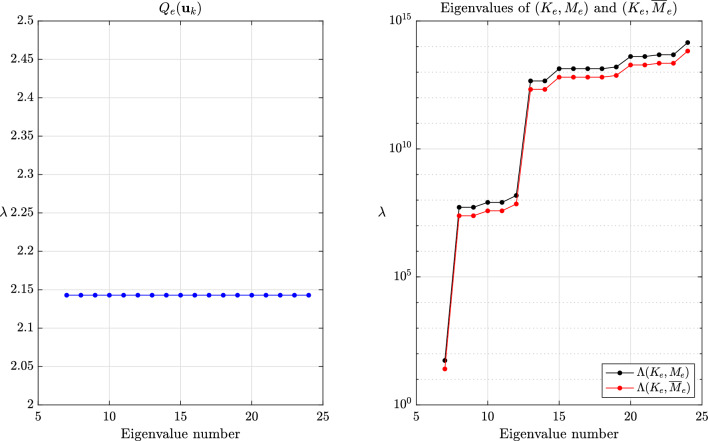
Values of $Q_e(\textbf{u}_k)$ for $k=7,\dots ,m$ and eigenvalues of $(K_e,M_e)$ and $(K_e,\overline{M}_e)$ for $\beta =1$ and the method of Olovsson et al.

**Fig. 3:**
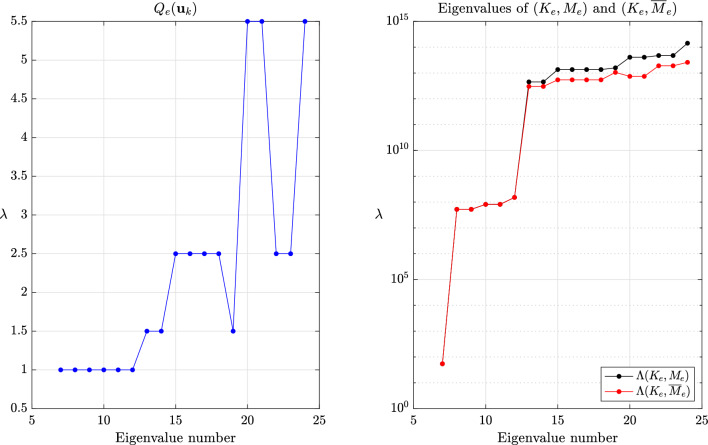
Values of $Q_e(\textbf{u}_k)$ for $k=7,\dots ,m$ and eigenvalues of $(K_e,M_e)$ and $(K_e,\overline{M}_e)$ for $\beta =1$ and the method of Hoffmann et al.

**Fig. 4:**
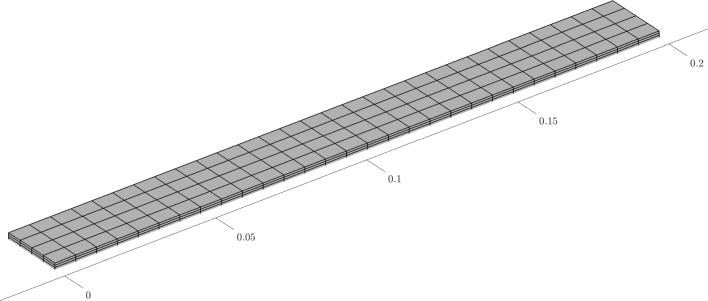
Hexahedral finite element mesh

### Ad hoc local SMS

The global behavior of the methods of Olovsson et al. [1] and Hoffmann et al. [35] is closely tied to the element constructions. For illustrating it, we first consider a single thin linear hexahedral element of size $1 \times 1 \times 10^{-3}$, whose Young modulus, Poisson ratio and density are $E=207$ GPa, $\nu =0.3$ and $\rho =7800$ kg/m^3^, respectively. The element (lumped) mass matrix $M_e$ is a scalar matrix such that the eigenvectors of $(K_e,M_e)$ are simply eigenvectors of the stiffness matrix. For the method of Olovsson et al., all eigenvectors $\textbf{u}_k$ for $k=7,8,\dots ,m$ (i.e. $\textbf{u}_k \in \mathbb {R}^m \setminus \ker (K_e)$) are also generalized eigenvectors of $(\overline{M}_e, M_e)$ since they are in the kernel of $I_3 \otimes \textbf{e}\textbf{e}^T$. The associated eigenvalues are $1+\frac{8}{7}\beta$ according to eq. (2.11) and it follows from Lemma 2.12 that the eigenvalues of the scaled matrix pair are

$$\begin{aligned}  &   \lambda _{i_k}(K_e,\overline{M}_e) = \frac{\lambda _k(K_e,M_e)}{Q_e(\textbf{u}_k)} = \frac{\lambda _k(K_e,M_e)}{1+\frac{8}{7}\beta }\\  &   \text {where} \quad Q_e(\textbf{x})=\frac{\textbf{x}^T \overline{M}_e \textbf{x}}{\textbf{x}^T M_e \textbf{x}} \end{aligned}$$

is the Rayleigh quotient. In this case, the perturbation scales down all eigenvalues uniformly and therefore preserves the eigenvalue ordering (i.e. $i_k=k$) for $k \ge 7$. These results are shown graphically in Fig. 2 for $\beta =1$ and confirm our theoretical findings. The results are far more interesting when considering the method of Hoffmann et al. Also in this case it turns out all eigenvectors of $(K_e,M_e)$ are eigenvectors of $(\overline{M}_e,M_e)$. The deep understanding of the deformation modes from engineering practice certainly guided the construction of the scaling matrix. These modes bear different names and are carefully listed and grouped in [27, Figure 3]. Applying Lemma 2.12 once again, we deduce that

$$\begin{aligned} \lambda _{i_k}(K_e,\overline{M}_e) = \frac{\lambda _k(K_e,M_e)}{Q_e(\textbf{u}_k)}, \end{aligned}$$

where the overline now obviously refers to scaled quantities for the method of Hoffmann et al. In this case, $Q_e(\textbf{u}_k)$ may take any value among those listed in eq. (2.15). Therefore, the scaling may not preserve the eigenvalue ordering. The values of $Q_e(\textbf{u}_k)$ for $k=7,\dots ,m$ are shown in Fig. 3 for $\beta =1$ alongside the eigenvalues of the scaled and original matrix pairs. It appears that $\lambda _{i_{20}}(K_e,\overline{M}_e)=\lambda _{i_{21}}(K_e,\overline{M}_e) < \lambda _{i_{19}}(K_e,\overline{M}_e)$ and confirms that the scaling does not preserve the eigenvalue ordering. Generally speaking, comparing the *k*th largest eigenvalues for the original and scaled matrix pairs is only relevant for “small” perturbations. In other cases, one should better understand how the original eigenvalues are transformed.

**Fig. 5:**
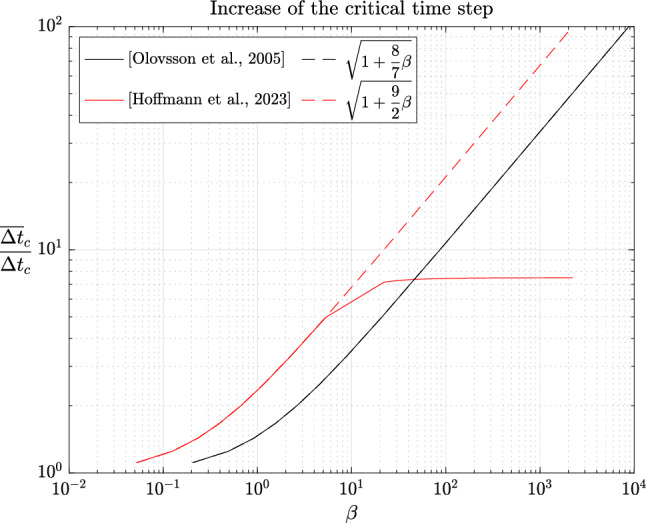
Increase of the critical time step for the methods of Olovsson et al. and Hoffmann et al.

The insights drawn locally will prove useful for illustrating some global properties. As benchmark, we consider a thin metal strip of length $\times$ width $\times$ thickness dimensions $200 \times 20 \times 2$ mm, whose Young modulus, Poisson ratio and density are again $E=207$ GPa, $\nu =0.3$ and $\rho =7800$ kg/m^3^, respectively. The plate is discretized with $40 \times 5 \times 4$ nodes in each respective dimension, producing 348 linear hexahedral elements. The resulting mesh is shown in Fig. 4. In this example, we test the sharpness of the bounds in Corollaries 2.22 and 2.24 for the methods of Olovsson et al. [1] and Hoffmann et al. [35], respectively. The increase of the critical time step is displayed in Fig. 5 along with its upper bound for different values of $\beta$, chosen such that the critical time step increases by (at most) a factor 1 to 100. For the method of Olovsson et al., the upper bound is very sharp over the entire range of values tested, whereas for the method of Hoffmann et al., the upper bound is satisfactory only for moderate values of $\beta$. For larger values, the ratio flattens out and the critical time step most likely becomes constrained by other deformation modes, not targeted by the method of Hoffmann et al. Nevertheless, both methods may achieve a similar moderate increase of the critical time step, albeit for different values of $\beta$.

**Fig. 6:**
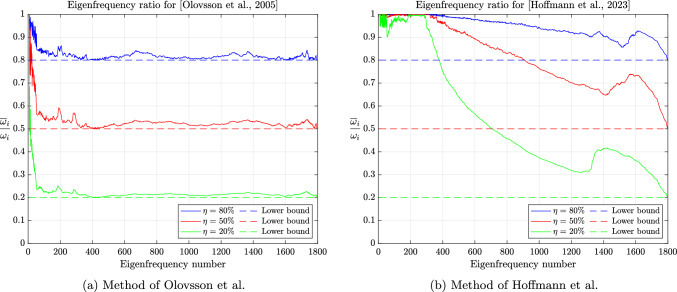
Eigenfrequency ratio for different frequency reduction targets

Following standard practice, Fig. 6 show the ratio of scaled over unscaled frequencies for selected frequency reduction targets $\eta$. For both methods, the values of $\beta$ are chosen such that the frequency ratio never falls below the target (either $80\%$, $50\%$ or $20\%$), which is guaranteed by the bounds of Corollaries 2.22 and 2.24. Note that the upper bounds of Corollaries 2.22 and 2.24 (valid over the entire spectrum) become lower bounds in this case. Interestingly, the overall trend reported in Fig. 6 is reminiscent of the local construction and the targeted modes. Admittedly, except preserving perhaps the first couple of eigenfrequencies, the method of Olovsson et al. seems quite inaccurate and Fig. 6a fuels existing concerns raised in [24, 35]. On the contrary, the method of Hoffmann et al. seems vastly superior, achieving a similar increase of the critical time step while yielding greater accuracy of the lower frequencies. However, if the scaling parameter exceeds a certain threshold, the increase of the critical time step stalls and instead the accuracy of the lower frequencies rapidly deteriorates. Indeed, the anomalies in the low-frequency spectrum for $\eta =20\%$ roughly appear at the same time the ratio of critical time steps departs from its upper bound in Fig. 5.

**Fig. 7:**
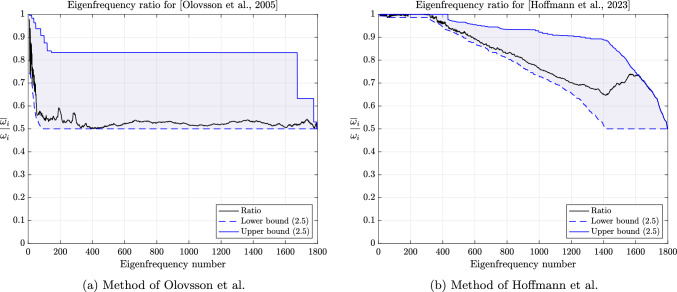
Bounds of eq. (2.5) for a frequency reduction target of $50\%$

Unfortunately, Corollaries 2.22 and 2.24 alone do not capture the selective nature of the methods since the eigenfrequency ratio is bounded uniformly, independently of whether the frequencies are large or small. However, the improved bounds (2.5) derived from the proof of Theorem 2.13 provide valuable insight on the matter. We have numerically computed those bounds in Fig. 7 for a frequency reduction target of $50\%$. They capture the eigenfrequency ratio reasonably well, especially at the extremes of the spectrum but are unfortunately difficult and expensive to compute. Nevertheless, they simplify considerably for the smallest and largest eigenvalue:

$$\begin{aligned}  &   \lambda _1(\overline{M},M) \le \frac{\lambda _1(K,M)}{\lambda _1(K,\overline{M})} \le Q(\overline{\textbf{u}}_1) \quad \text {and} \quad \\  &   Q(\overline{\textbf{u}}_n) \le \frac{\lambda _n(K,M)}{\lambda _n(K,\overline{M})} \le \lambda _n(\overline{M},M) \end{aligned}$$

where $\overline{\textbf{u}}_1$ and $\overline{\textbf{u}}_n$ denote the eigenvectors associated to the smallest and largest eigenvalues of $(K,\overline{M})$, respectively. Ideally, $Q(\overline{\textbf{u}}_1)$ should be as close to 1 as possible while $Q(\overline{\textbf{u}}_n)$ should maximize the Rayleigh quotient. These bounds again highlight the central role Theorem 2.13 plays in the theory.

**Fig. 8:**
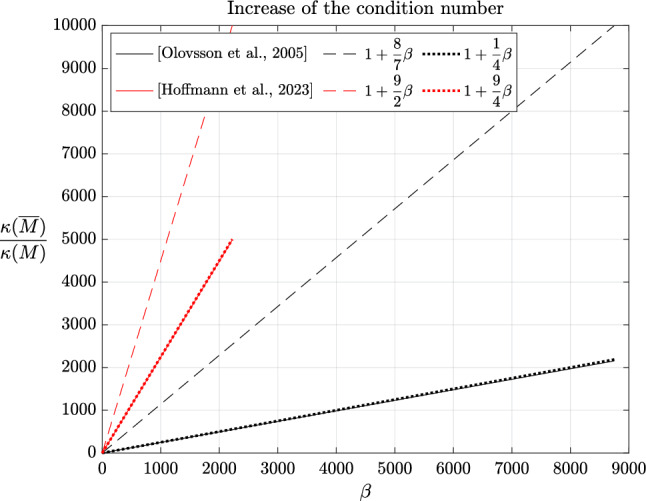
Increase of the condition number for the methods of Olovsson et al. and Hoffmann et al. for a uniform discretization with uniform material properties

If linear systems with the scaled mass matrix are solved iteratively, as advocated in [45], its condition number is often monitored. The upper bounds in Corollaries 2.22 and 2.24 suggest an increase in the number of iterations of iterative solvers (e.g. Conjugate Gradients) as the scaling parameter grows larger and may potentially offset the saving in the number of time steps. Fortunately, as shown in Fig. 8, the upper bounds seem quite pessimistic in this case. Although the growth is indeed linear, the rate is smaller than predicted. The numerically observed rates are shown in dotted line. The reason for the discrepancy between the theoretical rates and the observed ones is quite subtle and is due to a step in the proof of Lemma 2.16. Namely, the bounds $1 \le \Vert \textsf{L}\textbf{x}\Vert _2^2 \le p_{\max }$ may be loose for specific choices of $\textbf{x}$. For the method of Olovsson et al., one may derive a refined bound in the asymptotic regime as $\beta \rightarrow \infty$. For this purpose, we recall that for a lumped mass matrix *M* with uniform elements and material density, $\kappa (M)=p_{\max }$, where $p_{\max } \in \mathbb {N}^*$ is the maximum number of elements to which a node is connected. In our example, $p_{\max }=8$ and is attained for interior nodes. Furthermore, from the proof of Lemma 2.16, the following bounds hold:

$$\begin{aligned} \min _e \lambda _1(\overline{M}_e) \Vert \textsf{L}\textbf{x}\Vert _2^2 \le \textbf{x}^T \overline{M}\textbf{x} \le \max _e \lambda _m(\overline{M}_e) \Vert \textsf{L}\textbf{x}\Vert _2^2, \end{aligned}$$

where

$$\begin{aligned} \overline{M}=\sum _{e=1}^N L_e^T\overline{M}_eL_e, \quad \overline{M}_e = I_3 \otimes \overline{\textrm{M}}_e \end{aligned}$$

and

$$\begin{aligned} \overline{\textrm{M}}_e= &   \textrm{M}_e+\textrm{E}_e=\gamma _e((7+8\beta )I_8-\beta \textbf{e}\textbf{e}^T)\\  = &   \gamma _e(7 I_8 + \beta (8I_8-\textbf{e}\textbf{e}^T)). \end{aligned}$$

As previously noted, $\textrm{E}_e = \gamma _e\beta (8I_8-\textbf{e}\textbf{e}^T) \succeq 0$ and its norm increases with $\beta$. Therefore, as $\beta \rightarrow \infty$, the eigenvector $\textbf{x}_1$ associated to the smallest eigenvalue $\lambda _1(\overline{M})$ will try to annihilate this term. Since $\textbf{e} \in \ker (E_e)$ and $L_e\textbf{x}$ simply extracts entries from $\textbf{x}$, we expect $\textbf{x}_1$ to converge to the (normalized) vector of all ones; i.e. $\textbf{x}_1 =\frac{1}{\sqrt{n}}\textbf{1}$. In this case,

$$\begin{aligned} \Vert \textsf{L}\textbf{x}_1\Vert _2^2 = \frac{1}{n} \sum _{i=1}^n p_i = \frac{1}{n} \sum _{e=1}^N \sum _{j=1}^m 1 = \frac{mN}{n} \end{aligned}$$

reduces to the average connectivity, where *N* is the number of elements, *m* is the number of degrees of freedom per element and *n* is the total number of degrees of freedom. Finally, putting all the pieces back together,

$$\begin{aligned} \frac{\kappa (\overline{M})}{\kappa (M)}  &   \le \frac{\max _e \lambda _m(\overline{M}_e) }{\min _e \lambda _1(\overline{M}_e) \Vert \textsf{L}\textbf{x}_1\Vert _2^2} \\  &   \lesssim \frac{n}{mN} \frac{7+8\beta }{7} \frac{\max _e \gamma _e}{\min _e \gamma _e} = \frac{n}{mN}\left( 1 + \frac{8}{7}\beta \right) . \end{aligned}$$

Substituting the discretization parameters, we obtain $\frac{8n}{7mN} \approx 0.2463$, which is very close to the observed rate of 0.25. Our findings also consistently match the observations of Olovsson et al. [45], where the (non-normalized) condition number $\kappa (\overline{M})$ increased by roughly $1+2 \beta$. However, this rate is only expected to hold asymptotically and under the previously stated assumptions. The theoretically sound upper bound of $1+\frac{8}{7}\beta$ was much sharper on non-uniform meshes, suggesting that the behavior of the condition number is strongly problem-dependent. Performing a similar analysis for the method of Hoffmann et al. is far more involved and does not seem obvious at this stage.

### Local deflation

We consider the same benchmark as in the previous section but employ the local deflation techniques presented in Section  2.2.3. These techniques scale the element matrix pairs $(K_e,M_e)$ by simply applying Definition 2.10 locally; i.e.

$$\begin{aligned} \overline{K}_e&= K_e+V_ef(D_{e,2})V_e^T, \\ \overline{M}_e&= M_e+V_eg(D_{e,2})V_e^T, \end{aligned}$$

where $V_e=M_e U_{e,2}$, $D_{e,2}$ is the diagonal matrix containing the *r* largest eigenvalues of $(K_e,M_e)$ and $U_{e,2}$ contains the associated eigenvectors along its columns. Two specific choices of functions *f* and *g* where considered in Section  2.2.3:

$$\begin{aligned}  &   \text {Strategy 1: } {\left\{ \begin{array}{ll} f(\lambda )=0, &  \\ g(\lambda )=\alpha , \end{array}\right. } \qquad \\    &   \text {Strategy 2: } {\left\{ \begin{array}{ll} f(\lambda )=0, &  \\ g(\lambda )=\frac{\lambda }{\lambda _{m-r}(K_e,M_e)}-1, \end{array}\right. } \end{aligned}$$

where $\alpha >0$ is a scaling parameter. In both cases the stiffness is unaltered ($\overline{K}_e=K_e$) and the eigenvalues of the scaled matrix pairs $(\overline{K}_e, \overline{M}_e)$ are (see Theorem 2.11)

$$\begin{aligned}&\text {Strategy 1: } \lambda _{i_k}(\overline{K}_e, \overline{M}_e)\\  &\quad = {\left\{ \begin{array}{ll} \lambda _k(K_e,M_e) &  \text { for } k=1,\dots ,m-r, \\ \frac{\lambda _k(K_e,M_e)}{1+\alpha } &  \text { for } k=m-r+1,\dots ,m. \end{array}\right. } \\&\text {Strategy 2: } \lambda _k(\overline{K}_e, \overline{M}_e)\\  &\quad = {\left\{ \begin{array}{ll} \lambda _k(K_e,M_e) &  \text { for } k=1,\dots ,m-r, \\ \lambda _{m-r}(K_e,M_e) &  \text { for } k=m-r+1,\dots ,m. \end{array}\right. } \end{aligned}$$

Once again, local properties provide valuable insight on the global behavior and we consider for the time being a single thin linear hexahedral element of size $1 \times 1 \times 10^{-3}$ with the same material properties as the previous example. By construction, the matrix pairs $(K_e,M_e)$ and $(\overline{M}_e,M_e)$ share the same eigenvectors and Lemma 2.12 applies. Note, however, that the first strategy may not preserve the eigenvalue ordering ($i_k \ne k)$. This happens whenever $\alpha$ is large relative to the eigenvalue gap:

3.1 $$\begin{aligned} \alpha > \frac{\lambda _{m-r+1}(K_e,M_e)}{\lambda _{m-r}(K_e,M_e)}-1. \end{aligned}$$

Consequently, for the first strategy, the largest eigenvalue of the scaled matrix pair is

$$\begin{aligned} \lambda _m(\overline{K}_e, \overline{M}_e) = \max \left\{ \lambda _{m-r}(K_e,M_e), \ \frac{\lambda _m(K_e,M_e)}{1+\alpha }\right\} . \end{aligned}$$

**Fig. 9:**
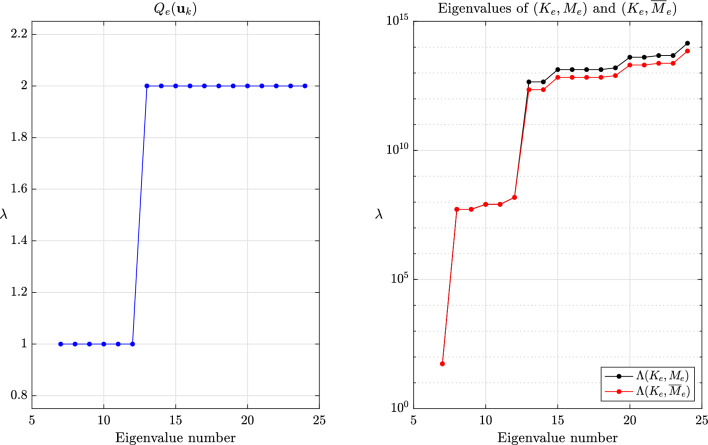
Values of $Q_e(\textbf{u}_k)$ for $k=7,\dots ,m$ and eigenvalues of $(K_e,M_e)$ and $(K_e,\overline{M}_e)$ for the first strategy with $r=12$ and $\alpha =1$

**Fig. 10:**
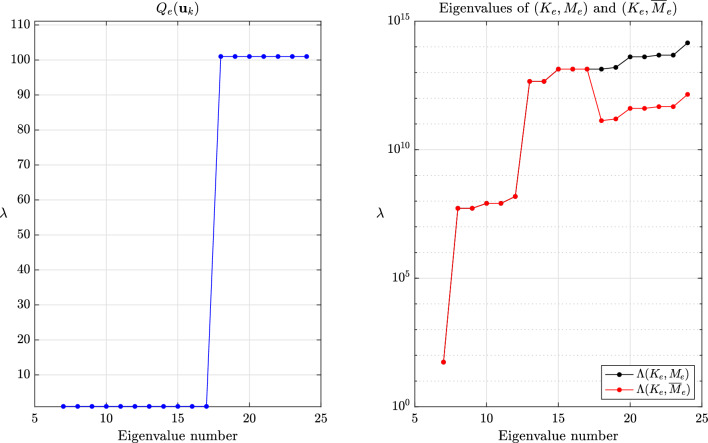
Values of $Q_e(\textbf{u}_k)$ for $k=7,\dots ,m$ and eigenvalues of $(K_e,M_e)$ and $(K_e,\overline{M}_e)$ for the first strategy with $r=7$ and $\alpha =10^2$

Thus, as $\alpha$ gets increasingly large, the largest eigenvalue is given by $\lambda _{m-r}(K_e,M_e)$, which no longer depends on $\alpha$ but only on the deflation rank and therefore places a threshold on the maximum increase of the critical time step. Figures  9 and 10 show two different cases where the gap assumption eq. (3.1) is satisfied and violated, respectively. For the second example, one also notices that $\lambda _m(\overline{K}_e, \overline{M}_e)=\lambda _{m-r}(K_e,M_e)$ (Fig. 10).

**Fig. 11:**
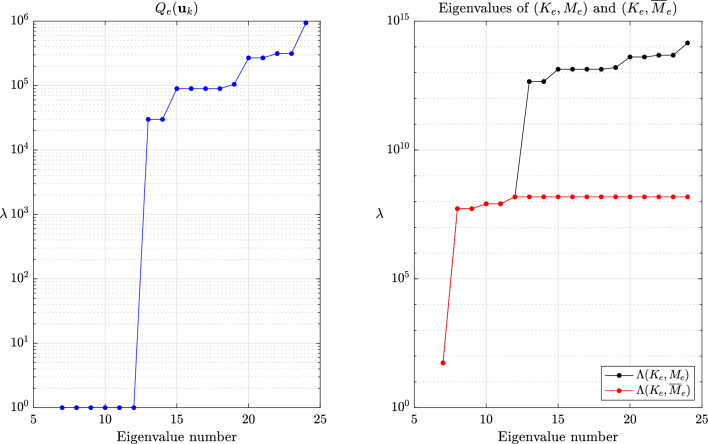
Values of $Q_e(\textbf{u}_k)$ for $k=7,\dots ,m$ and eigenvalues of $(K_e,M_e)$ and $(K_e,\overline{M}_e)$ for the second strategy with $r=12$

Choosing a large value of $\alpha$ may quickly undermine the quality of the solution as it moves inaccurate high frequency modes to the low-frequency regime. González and Park [25] already highlighted the limitations of this technique for a simple bar model and its alarming impact on the accuracy. Thus, one should exercise caution when using this technique. The second strategy, on the contrary, always preserves the eigenvalue ordering. The result, illustrated in Fig. 11 for $r=12$, consists in shaving off the upper part of the spectrum.

**Fig. 12:**
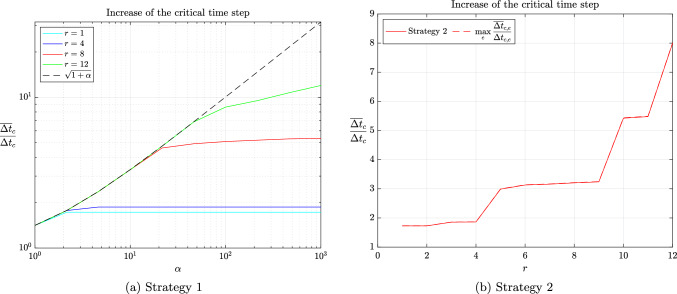
Increase of the critical time step for local deflation strategies

We now investigate the global properties of the method using the same finite element mesh and material properties as in the previous section. Figure 12a shows the increase of the critical time step for the first strategy for fixed rank values and increasing values of $\alpha$ together with the upper bound from Corollary 2.19. Interestingly, Fig. 12a displays a threshold effect reminiscent of the local behavior. Figure 12b shows the increase of the critical time step for the second strategy, which is perfectly captured by the upper bound of Corollary 2.20.

**Fig. 13:**
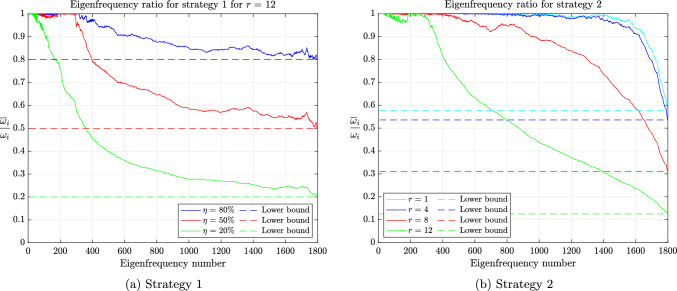
Eigenfrequency ratio

The eigenfrequency ratio for the entire spectrum is shown in Fig. 13a for the first strategy and in Fig. 13b for the second one. The first strategy features two parameters: the deflation rank *r* and the scaling parameter $\alpha$. Thus, Fig. 13a shows the eigenfrequency ratio for a fixed deflation rank ($r=12$) and selected frequency reduction targets $\eta$. Once again, the scaling parameter $\alpha$ is computed such that the eigenfrequency ratio never falls below the target. On the contrary, Fig. 13b plots the eigenfrequency ratio for selected values of the deflation rank *r*, which is the only parameter for the second strategy.

Admittedly, the first strategy cannot significant increase the critical time step without compromising the accuracy of the lower frequencies. However, the second strategy for deflation ranks $r=1,\dots ,8$ seems quite promising as the eigenfrequency ratio abruptly decays only towards the end of the spectrum, ensuring both good accuracy and a significant increase of the critical time step. Finally, we note that the proof strategy of Corollaries 2.22 and 2.24 also immediately yields (not necessarily tight) upper bounds on the condition number of the scaled mass matrix for these methods.

### Summary

The ad hoc mass scaling strategies of Olovsson et al. and Hoffmann et al. may be viewed as inexact local deflation strategies based on low-frequency modes. One notable advantage of using low-frequency modes instead of high-frequency ones is that the former includes rigid-body modes and are known regardless of how distorted the element is. Thus, they offer better guarantees of robustness. Moreover, engineers have developed a very good understanding of the deformation mode shapes for an hexahedral element (see [27, Figure 3]), based on which they constructed these ad hoc strategies. While the method of Hoffmann et al. seems significantly better than the one of Olovsson et al., it still does not exactly target the right modes, which both impedes on the accuracy and the increase of the critical time step. Preserving the eigenvalue ordering seems essential to avoid interchanging modes, which might disastrously affect the accuracy of the solution. Exact local deflation strategies may deliver far better results but assume that the stiffness matrix is explicitly available, which is rarely the case in commercial software, especially for nonlinear problems. We believe it might be possible to further improve the method of Hoffmann et al. for obtaining the desired behavior but leave this task to the engineering community.

The experiments have also revealed that the global properties are tightly connected to the local ones. The global matrix pairs (*K*, *M*) and $(M,\overline{M})$ may nearly share some eigenspaces but we have not explored this hypothesis any further.

## Conclusion

Selective mass scaling techniques are increasingly popular in structural dynamics for increasing the critical time step of explicit time integration methods, while preserving the accuracy of structurally important lower frequencies and mode shapes. While physical intuition is sometimes sufficient for identifying and eliminating the constraint on the step size, we have substantiated it with a strong theoretical analysis thereby unraveling appealing properties of some of the most popular methods. In particular, we have drawn a unifying picture and provided a clearer positioning of the methods by bridging the gap between different communities and highlighting fundamental similarities and differences. Practically speaking though, while some methods are certainly valuable to the engineering community, others are mostly impractical and often based on elementary linear algebra facts known for decades. Moreover, nearly all methods lead to a non-diagonal mass matrix and defeat the spirit of explicit dynamics. The methods are only competitive if the saving in the number of time steps overtakes the additional cost per time step for repeatedly solving linear systems with the scaled mass matrix. Directly modifying the inverse mass matrix [26–28, 37, 42] or the stiffness matrix [19] suggests itself as a promising pathway. Finally, the benefits and drawbacks of mass scaling must be carefully weighted on a case-by-case basis. While mass scaling may certainly help in some cases, it might also irremediably degrade the solution, depreciating advanced material models and finite elements if not undermining the very purpose of numerical simulations. We feel this issue is largely overlooked, especially for nonlinear problems, and advise caution when using such methods.

## Footnotes

1 Later authors [32, 33] have defined instead $\textrm{E}_e = \frac{\beta m_e}{8}(I_8-\textbf{u}\textbf{u}^T)$ but we will stick to the original formulation, as proposed in [1], where $\beta =1$ doubles the diagonal entries of $M_e$. This small subtlety merely changes a constant.

2 The $\sqrt{1+\beta }$ factor increase holds for alternative formulations presented in [32, 33]. Note the striking similarity with Corollary 2.19.

## References

[1] Olovsson L, Simonsson K, Unosson M (2005) Selective mass scaling for explicit finite element analyses. Int J Numer Meth Eng 63(10):1436–1445

[2] Anitescu C, Nguyen C, Rabczuk T, Zhuang X (2019) Isogeometric analysis for explicit elastodynamics using a dual-basis diagonal mass formulation. Comput Methods Appl Mech Eng 346:574–591

[3] Nguyen T-H, Hiemstra RR, Eisenträger S, Schillinger D (2023) Towards higher-order accurate mass lumping in explicit isogeometric analysis for structural dynamics. Comput Methods Appl Mech Eng 417:116233

[4] Voet Y, Sande E, Buffa A (2023) A mathematical theory for mass lumping and its generalization with applications to isogeometric analysis. Comput Methods Appl Mech Eng 410:116033

[5] Li X, Wang D (2022) On the significance of basis interpolation for accurate lumped mass isogeometric formulation. Comput Methods Appl Mech Eng 400:115533

[6] Hughes TJ (2012) The finite element method: linear static and dynamic finite element analysis. Courier Corporation

[7] Bathe K-J (2006) Finite element procedures. Klaus-Jurgen Bathe

[8] Gallistl D, Huber P, Peterseim D (2017) On the stability of the Rayleigh-Ritz method for eigenvalues. Numer Math 137:339–351

[9] Gahalaut K, Tomar S (2012) Condition number estimates for matrices arising in the isogeometric discretizations. Tech. rep, Johann Radon Institut (RICAM)

[10] Hughes TJ, Cottrell JA, Bazilevs Y (2005) Isogeometric analysis: CAD, finite elements, NURBS, exact geometry and mesh refinement. Comput Methods Appl Mech Eng 194(39–41):4135–4195

[11] Cottrell JA, Reali A, Bazilevs Y, Hughes TJ (2006) Isogeometric analysis of structural vibrations. Comput Methods Appl Mech Eng 195(41–43):5257–5296

[12] Cottrell JA, Hughes T, Reali A (2007) Studies of refinement and continuity in isogeometric structural analysis. Comput Methods Appl Mech Eng 196(41–44):4160–4183

[13] Hughes TJ, Evans JA, Reali A (2014) Finite element and NURBS approximations of eigenvalue, boundary-value, and initial-value problems. Comput Methods Appl Mech Eng 272:290–320

[14] Manni C, Sande E, Speleers H (2022) Application of optimal spline subspaces for the removal of spurious outliers in isogeometric discretizations. Comput Methods Appl Mech Eng 389:114260

[15] Manni C, Sande E, Speleers H (2023) Outlier-free spline spaces for isogeometric discretizations of biharmonic and polyharmonic eigenvalue problems. Comput Methods Appl Mech Eng 417:116314

[16] Hiemstra RR, Hughes TJ, Reali A, Schillinger D (2021) Removal of spurious outlier frequencies and modes from isogeometric discretizations of second-and fourth-order problems in one, two, and three dimensions. Comput Methods Appl Mech Eng 387:114115

[17] Deng Q, Calo VM (2021) A boundary penalization technique to remove outliers from isogeometric analysis on tensor-product meshes. Comput Methods Appl Mech Eng 383:113907

[18] Nguyen T-H, Hiemstra RR, Stoter SK, Schillinger D (2022) A variational approach based on perturbed eigenvalue analysis for improving spectral properties of isogeometric multipatch discretizations. Comput Methods Appl Mech Eng 392:114671

[19] Voet Y, Sande E, Buffa A (2024) Mass lumping and outlier removal strategies for complex geometries in isogeometric analysis, arXiv preprint arXiv:2402.14956

[20] Key SW, Beisinger ZE (1971) The transient dynamic analysis of thin shells by the finite element method. In: Proceedings of the third conference on matrix methods in structural mechanics

[21] Hughes TJ, Cohen M, Haroun M (1978) Reduced and selective integration techniques in the finite element analysis of plates. Nucl Eng Des 46(1):203–222

[22] Hotelling H (1943) Some new methods in matrix calculation. Ann Math Stat 14(1):1–34

[23] Macek RW, Aubert BH (1995) A mass penalty technique to control the critical time increment in explicit dynamic finite element analyses. Earthq Eng Struct Dyn 24(10):1315–1331

[24] Tkachuk A, Bischoff M (2014) Local and global strategies for optimal selective mass scaling. Comput Mech 53(6):1197–1207

[25] González JA, Park K (2020) Large-step explicit time integration via mass matrix tailoring. Int J Numer Meth Eng 121(8):1647–1664

[26] Olovsson L, Unosson M, Simonsson K (2004) Selective mass scaling for thin walled structures modeled with tri-linear solid elements. Comput Mech 34:134–136

[27] Cocchetti G, Pagani M, Perego U (2013) Selective mass scaling and critical time-step estimate for explicit dynamics analyses with solid-shell elements. Comput Struct 127:39–52

[28] Cocchetti G, Pagani M, Perego U (2015) Selective mass scaling for distorted solid-shell elements in explicit dynamics: optimal scaling factor and stable time step estimate. Int J Numer Meth Eng 101(9):700–731

[29] Oesterle B, Trippmacher J, Tkachuk A, Bischoff M (2022) Intrinsically selective mass scaling with hierarchic structural element formulations, in: Proceedings of the YIC 2021-VI ECCOMAS Young Investigators Conference, Editorial Universitat Politècnica de València, pp. 99–108

[30] Oesterle B, Tkachuk A, Bischoff M (2023) Finite element technology-based selective mass scaling for shear deformable structural element formulations, in: 9th International Conference on Computational Methods in Structural Dynamics and Earthquake Engineering, COMPDYN 2023 pp 1965–1973

[31] Krauß L-M, Thierer R, Bischoff M, Oesterle B (2024) Intrinsically selective mass scaling with hierarchic plate formulations. Comput Methods Appl Mech Eng 432:117430

[32] Gavoille S (2013) Enrichissement modal du selective mass scaling. In: 11e colloque national en calcul des structures

[33] Borrvall T (2011) Selective mass scaling (SMS): Theory and Practice

[34] Morancay L, Winkelmuller G (2009) Dynamic condensation and selective mass scaling in RADIOSS Explicit, in: CFM 2009-19ème Congrès Français de Mécanique, AFM, Maison de la Mécanique, 39/41 rue Louis Blanc-92400 Courbevoie,

[35] Hoffmann M, Tkachuk A, Bischoff M, Oesterle B (2023) Finite element technology-based selective mass scaling for explicit dynamic analyses of thin-walled structures using solid elements, in: 9th International Conference on Computational Methods in Structural Dynamics and Earthquake Engineering, COMPDYN 2023, pp 1974–1982

[36] Ye W, Bel-Brunon A, Catheline S, Rochette M, Combescure A (2017) A selective mass scaling method for shear wave propagation analyses in nearly incompressible materials. Int J Numer Meth Eng 109(2):155–173

[37] González JA, Kolman R, Cho S, Felippa C, Park K (2018) Inverse mass matrix via the method of localized Lagrange multipliers. Int J Numer Meth Eng 113(2):277–295

[38] Eisenträger S, Radtke L, Garhuom W, Löhnert S, Düster A, Juhre D, Schillinger D (2024) An eigenvalue stabilization technique for immersed boundary finite element methods in explicit dynamics. Comput Math Appl 166:129–168

[39] Leidinger L (2020) Explicit isogeometric B-Rep analysis for nonlinear dynamic crash simulations, Ph.D. thesis, Technische Universität München

[40] Stoter SK, Divi SC, van Brummelen EH, Larson MG, de Prenter F, Verhoosel CV (2023) Critical time-step size analysis and mass scaling by ghost-penalty for immersogeometric explicit dynamics. Comput Methods Appl Mech Eng 412:116074

[41] Tkachuk A, Bischoff M (2013) Variational methods for selective mass scaling. Comput Mech 52:563–570

[42] Tkachuk A, Bischoff M (2015) Direct and sparse construction of consistent inverse mass matrices: general variational formulation and application to selective mass scaling. Int J Numer Meth Eng 101(6):435–469

[43] Schaeuble A-K, Tkachuk A, Bischoff M (2017) Variationally consistent inertia templates for B-spline-and NURBS-based FEM: inertia scaling and customization. Comput Methods Appl Mech Eng 326:596–621

[44] González JA, Kopačka J, Kolman R, Cho S, Park K (2019) Inverse mass matrix for isogeometric explicit transient analysis via the method of localized Lagrange multipliers. Int J Numer Meth Eng 117(9):939–966

[45] Olovsson L, Simonsson K (2006) Iterative solution technique in selective mass scaling. Commun Numer Methods Eng 22(1):77–82

[46] Stoter SK, Nguyen T-H, Hiemstra RR, Schillinger D (2022) Variationally consistent mass scaling for explicit time-integration schemes of lower-and higher-order finite element methods. Comput Methods Appl Mech Eng 399:115310

[47] Hartmann S, Benson DJ (2015) Mass scaling and stable time step estimates for isogeometric analysis. Int J Numer Meth Eng 102(3–4):671–687

[48] Adam C, Bouabdallah S, Zarroug M, Maitournam H (2015) Stable time step estimates for NURBS-based explicit dynamics. Comput Methods Appl Mech Eng 295:581–605

[49] Flanagan D, Belytschko T (1984) Eigenvalues and stable time steps for the uniform strain hexahedron and quadrilateral. Journal of Applied Mechanics

[50] Varga RS (2011) Geršgorin and his circles. Springer, Berlin

[51] Schaeuble A-K, Tkachuk A, Bischoff M (2018) Time step estimates for explicit dynamics with reciprocal mass matrices. Comput Struct 202:74–84

[52] Papadrakakis M, Fragiadakis M (2019) (Eds.) Time step estimates for reciprocal mass matrices using Ostrowski’s bounds

[53] Hinton E, Rock T, Zienkiewicz O (1976) A note on mass lumping and related processes in the finite element method. Earthq Eng Struct Dyn 4(3):245–249

[54] Hallquist J (2005) LS-DYNA Theory manual, Livermore Software Technology Corporation

[55] Belytschko T, Mindle WL (1980) Flexural wave propagation behavior of lumped mass approximations. Comput Struct 12(6):805–812

[56] Stewart GW (1979) Pertubation bounds for the definite generalized eigenvalue problem. Linear Algebra Appl 23:69–85

[57] Stewart GW, Sun J-G (1990) Matrix perturbation theory. Academic Press, Computer science and scientific computing

[58] Parlett BN (1991) Symmetric matrix pencils. J Comput Appl Math 38(1–3):373–385

[59] Parlett BN (1998) The symmetric eigenvalue problem, SIAM,

[60] Saad Y (2011) Numerical methods for large eigenvalue problems: revised edition, SIAM,

[61] Ericsson T, Ruhe A (1980) The spectral transformation Lanczos method for the numerical solution of large sparse generalized symmetric eigenvalue problems. Math Comput 35(152):1251–1268

[62] Askes H, Nguyen DC, Tyas A (2011) Increasing the critical time step: micro-inertia, inertia penalties and mass scaling. Comput Mech 47:657–667

[63] Higham NJ (2008) Functions of matrices: theory and computation, SIAM,

[64] Horn RA, Johnson CR (2012) Matrix analysis. Cambridge University Press

[65] Fried I (1972) Bounds on the extremal eigenvalues of the finite element stiffness and mass matrices and their spectral condition number. J Sound Vib 22(4):407–418

[66] Wathen AJ (1987) Realistic eigenvalue bounds for the Galerkin mass matrix. IMA J Numer Anal 7(4):449–457

[67] Irons BM, Treharne G (1971) A bound theorem in eigenvalues and its practical applications, in: Proceedings of the Third Conference on Matrix Methods in Structural Mechanics, Wright Patterson Air Force Base, Ohio, 245–254

[68] Cottereau R, Sevilla R (2018) Stability of an explicit high-order spectral element method for acoustics in heterogeneous media based on local element stability criteria. Int J Numer Meth Eng 116(4):223–245

[69] Leidinger L, Breitenberger M, Bauer A, Hartmann S, Wüchner R, Bletzinger K-U, Duddeck F, Song L (2019) Explicit dynamic isogeometric B-Rep analysis of penalty-coupled trimmed NURBS shells. Comput Methods Appl Mech Eng 351:891–927

[70] Geuzaine C, Remacle J-F (2009) Gmsh: a 3-D finite element mesh generator with built-in pre-and post-processing facilities. Int J Numer Meth Eng 79(11):1309–1331

